# Complex Strain Scapes in Reconstructed Transition-Metal Dichalcogenide Moiré Superlattices

**DOI:** 10.1021/acsnano.3c00609

**Published:** 2023-04-06

**Authors:** Álvaro Rodríguez, Javier Varillas, Golam Haider, Martin Kalbáč, Otakar Frank

**Affiliations:** † J. Heyrovský Institute of Physical Chemistry, Czech Academy of Sciences, Dolejškova 2155/3, 182 23 Prague, Czech Republic; ‡ Materials Science Factory, Instituto de Ciencia de Materiales de Madrid, Consejo Superior de Investigaciones Científicas, 28049 Madrid, Spain; ¶ Institute of Thermomechanics, Czech Academy of Sciences, Dolejškova 1402/5, 182 00 Prague 8, Czech Republic

**Keywords:** Transition-Metal Dichalcogenide, Moiré Superlattice, Atomic Reconstruction, Raman Spectroscopy, Molecular Dynamics, Strain

## Abstract

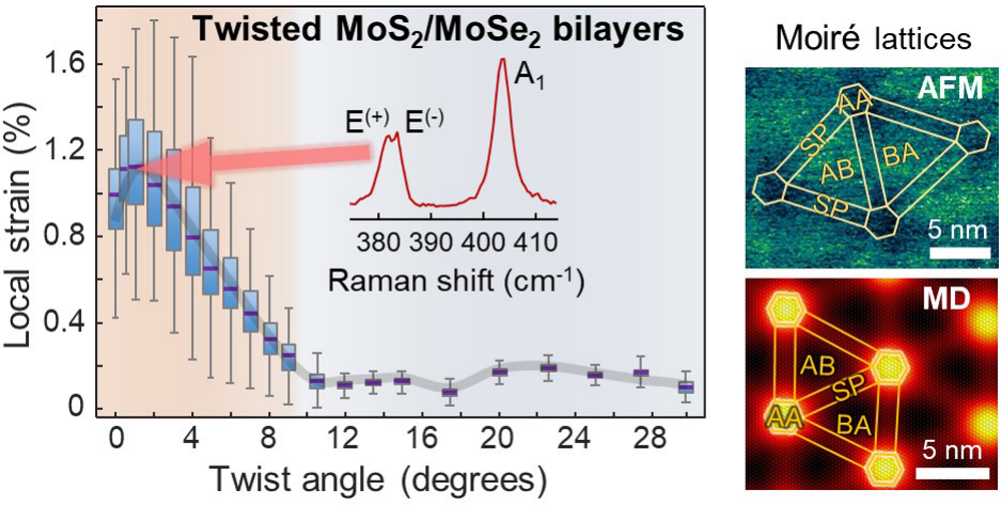

We investigate the intrinsic strain associated with the coupling of twisted MoS_2_/MoSe_2_ heterobilayers by combining experiments and molecular dynamics simulations. Our study reveals that small twist angles (between 0 and 2°) give rise to considerable atomic reconstructions, large moiré periodicities, and high levels of local strain (with an average value of ∼1%). Moreover, the formation of moiré superlattices is assisted by specific reconstructions of stacking domains. This process leads to a complex strain distribution characterized by a combined deformation state of uniaxial, biaxial, and shear components. Lattice reconstruction is hindered with larger twist angles (>10°) that produce moiré patterns of small periodicity and negligible strains. Polarization-dependent Raman experiments also evidence the presence of an intricate strain distribution in heterobilayers with near-0° twist angles through the splitting of the E_2g_^1^ mode of the top (MoS_2_) layer due to atomic reconstruction. Detailed analyses of moiré patterns measured by AFM unveil varying degrees of anisotropy in the moiré superlattices due to the heterostrain induced during the stacking of monolayers.

## Introduction

Two-dimensional (2D) materials have attracted much attention in recent years due to their tunable optoelectronic properties that can be tailored by different external perturbations^1^ and to the large amount of new and exotic features they exhibit when stacked in different combinations of multilayer 2D materials.^2,3^ Transition-metal dichalcogenides (TMDCs) are room-temperature semiconductors that can be exfoliated down to the monolayer thickness, exhibiting strong photoluminescence (PL).^4^ The extremely strong PL signal facilitates the identification of monolayers using standard optical characterization techniques. When combining two TMDC monolayers in vertical heterostructures, a charge transfer process occurs through the formation of a type-II band alignment.^5^ The twist angle, θ, between the two monolayers determines the optical and electronic properties of the resulting material.^6,7^ The recent emergence of twistronics based on the observation of superconductivity in twisted bilayer graphene (t-BLG)^8^ and moiré excitons in TMDC heterostructures^9−11^ has motivated the study of other 2D homo- and heterostructures. For example, the quantum anomalous Hall effect was observed in t-BLG aligned to hexagonal boron nitride (h-BN),^12^ and an incompressible Mott-like state of electrons was demonstrated in a long-period TMDC/h-BN moiré superlattice.^13^ In addition, ferroelectricity was observed in twisted h-BN^14^ and in TMDC homo- and heterobilayers.^15,16^

Small-angle twisted TMDC heterobilayers produce long-range-ordered moiré superlattices where the interlayer exciton exhibits a relatively strong room-temperature PL^17^ as well as more extravagant properties, such as sensitivity to circularly polarized light,^9^ the presence of multiple excitonic resonances,^10^ and long-lived excitons confined in the moiré potential.^18^ Moreover, atomic reconstructions of the hexagonal lattice were linked to the formation of flat bands in the electronic structure of TMDC bilayers.^19−21^

Moiré patterns can be visualized using high-resolution microscopy techniques, such as scanning transmission electron microscopy or scanning probe microscopy.^22−25^ In particular, the moiré patterns of TMDC heterobilayers were observed via piezoresponse force microscopy, Kelvin probe microscopy, and conductive atomic force microscopy.^26,27^ While several studies have successfully observed lattice reconstruction in moiré patterns of TMDC homo- and heterostructures,^22,26^ a fundamental understanding of the heterostrain occurring in the moiré superlattices is still missing in the literature. This knowledge is crucial for the evolution of the field.

For TMDCs, recent studies have reported on the effect of varying the twist angle on the strain levels manifested in MoS_2_/MoS_2_ homobilayers^28^ and in MoS_2_/WSe_2_ heterobilayers.^29^ Experimentally, changes in shape and shift of the Raman in-plane E mode were reported to be one of the indicators of the observed variations in strain. Through first-principles calculations, three regimes of atomic reconstruction were found in MoS_2_ bilayers, where small twist angles (between 2 and 5°) exhibited the highest atomic-level strains.^28^ However, the highest strains were observed with a twist angle of ∼0° in MoS_2_/WSe_2_ heterostructures.^29^ Such a difference in strains as a function of the twist angle is a consequence of the lattice mismatch concomitant to TMDC heterobilayers. Thus, the formation of moiré patterns is observed in heterobilayers at a 0° twist angle. In contrast, a small twist angle is required to form moiré superlattices in bilayers without a lattice mismatch, such as in homobilayers; see Supplementary Figure S1.

Heterostrain is defined as the relative strain of the top layer with respect to the bottom one, which is deemed to be introduced during the fabrication process.^30^ In t-BLG, it was found that the presence of small intralayer strains (between 0.1 and 0.7%)^30^ might lead to the emergence of flat bands and correlated states.^31−33^ In twisted MoS_2_ bilayers, the strain was considered to be intrinsic to the bilayer formation due to lattice reconstruction at certain twist angles.^28,34^ Furthermore, large uniaxial heterostrain levels were also shown to form one-dimensional moiré patterns in both twisted bilayer graphene and TMDC heterobilayers.^35−37^ Controlling and tuning the heterostrain is a viable option toward applications where anisotropy is required. For instance, interlayer PL of one-dimensional TMDC moiré superlattices can exhibit linear-polarization dependence as shown by Bai et al.^37^ and could be implemented in polarization-sensitive optoelectronic devices. It is still unclear to which extent these strains arise as a result of the strong interaction between the two layers or whether heterostrain states are also introduced during the stacking process.

The fabrication of perfectly aligned heterobilayers composed of mechanically exfoliated monolayers is challenging. A straightforward method to precisely control the twist angle between two different TMDC monolayers is currently lacking. Although second-harmonic generation spectroscopy is the most widespread technique to experimentally determine the twist angle, this approach has some uncertainties^38^ and is not accessible with standard optical characterization setups. Another characterization method that permits the quantitative measurement of a twist angle is highly desirable. Raman spectroscopy has traditionally been used to extract a plethora of useful information from a range of 2D materials. For instance, Raman measurements enable the determination of the number of layers in TMDC crystals, especially when analyzing the shear and layer-breathing modes in the low-frequency region.^39−41^ A similar approach was utilized for quantitatively assigning the twist angle in twisted WSe_2_ bilayers.^42^ Parzefall et al.^38^ reported on the correlation between the interlayer shear mode observed in the low-frequency Raman spectrum of MoSe_2_/WSe_2_ heterostructures and the atomic reconstruction taking place in the system, which essentially indicates a near-0° alignment. The dependence of the interlayer exciton emission (both energy and intensity) with the twist angle has also been observed for different TMDC heterobilayers.^43,44^

In the present work, we focus on MoS_2_/MoSe_2_ heterobilayers. The ensuing intralayer strain states in reconstructed MoS_2_/MoSe_2_ heterobilayers are examined via polarized Raman spectroscopy, AFM measurements, and molecular dynamics (MD) simulations. We show that the Raman in-plane E mode of MoS_2_ can be used as a fingerprint of lattice reconstruction and, hence, of the presence of a near-0° twist angle in MoS_2_/MoSe_2_ systems. Moreover, we extensively study the strain as a function of the twist angle by means of large-scale MD simulations. The analysis demonstrates the complexity of the strain distributions across the moiré patterns in MoS_2_/MoSe_2_ heterostructures and evidences that lattice reconstruction occurs at small twist angles. Finally, we prove that upon applying uniaxial prestraining to the MoS_2_ layer, the heterostrain that results from the formation of moiré patterns increases.

## Results and Discussion

### Optical and Surface Characterization of MoS_2_/MoSe_2_ Heterobilayers

We aim to study the strains occurring in reconstructed MoS_2_/MoSe_2_ heterobilayers, that is, when two monolayers are stacked at θ ≈ 0° producing a large-periodicity moiré superlattice. The samples were prepared by sequential transfer of mechanically exfoliated monolayers onto SiO_2_/Si substrates using a transfer stage with a controllable target rotation; see Experimental Methodology.

**Figure 1:**
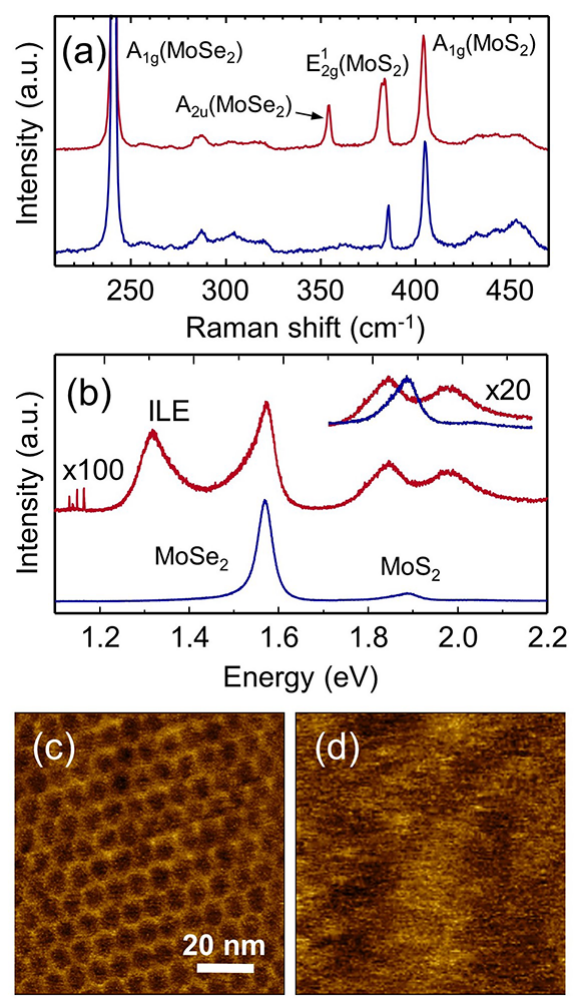
Optical and topographic characterization of twisted MoS_2_/MoSe_2_ heterobilayers. (a) Raman and (b) PL spectra of coupled (at θ ≈ 0°) (red) and uncoupled (blue) MoS_2_/MoSe_2_ bilayers. For simplicity, the notation of the Raman modes alludes to the homobilayer notation. AFM topography images of (c) coupled (at θ ≈ 0°) and (d) uncoupled MoS_2_/MoSe_2_ bilayers. The large moiré periodicity confirms the very small twist angle between the layers in the coupled heterobilayer.

Figure 1(a) shows the Raman spectra of a coupled MoS_2_/MoSe_2_ bilayer stacked at θ ≈ 0° (in red) and a bilayer formed by uncoupled monolayers (in blue). The MoS_2_ is on top in all cases. The coupling is induced by heating the sample above 80 °C, as evidenced by the reduction of the interlayer spacing (Supplementary Figure S2) and the strong intralayer PL quenching.^45^ The Raman spectrum of the uncoupled layers is just the combination of the noninteracting MoS_2_ and MoSe_2_ monolayers, whereas the spectrum of the coupled bilayer system (with θ ≈ 0°) exhibits a new mode emerging at ∼354 cm^–1^ that can be assigned to the A_2u_ mode of MoSe_2_. This mode becomes Raman active in the bilayer as predicted by group theory due to the reduction of symmetry in twisted heterostructures.^46^ Also, it can be considered a fingerprint of the bilayer coupling in twisted MoS_2_/MoSe_2_ heterobilayers, resembling the MoS_2_/WSe_2_ case from Chiu et al.^47^ We note that because the heterobilayer belongs to the *C*_3*v*_ point group, the correct notation for such a Raman mode should be A_1_.^46^ (See Supplementary Figure S6 in Section S3 for the extended assignment of the Raman modes.) In addition, the Raman spectrum of the coupled bilayer unveils another interesting peculiarity related to the E mode (E_2g_^1^ for homobilayers), which manifests a larger peak width than that found in the MoS_2_ monolayer; see Supplementary Figure S5 (Section S3) This mode is well-known to be sensitive to the in-plane strain. A similar broadening was observed when applying small uniaxial strains to MoS_2_ mono- and bilayers.^48,49^

The PL spectra of the same samples are shown in Figure 1(b). The PL emission of the bilayer stacked at θ ≈ 0° undergoes a significant quenching of the intensity of both MoS_2_ and MoSe_2_ intralayer excitons due to the interlayer charge transfer.^50^ When carefully comparing the energy of the MoS_2_ excitons, a redshift of both A and B excitons can be observed. This is consistent with the observed behavior of the Raman features, as the intralayer tensile strains are expected to decrease the band gap energy, thereby redshifting the energy of the excitons in MoS_2_.^51^ Another recognizable difference between the two samples is the appearance of a strong PL peak at ∼1.3 eV in the coupled bilayer. In agreement with previous reports,^52,53^ we assign this peak to the interlayer exciton emission. These optical measurements provide a firm signature of the strong interaction between the two layers in the coupled systems, as also evidenced in the AFM topography image shown in Figure 1(c). A moiré pattern with a periodicity of ∼8 nm is observed, which further corroborates the presence of a twist angle near 0°. We find such moiré patterns and Raman features in a set of five aligned MoS_2_/MoSe_2_ samples; see Supplementary Figure S3 (Section S3). Additional AFM images of larger areas are also provided in Supplementary Figure S7 (Section S3). Note that in Figure 1(d), the moiré patterns are absent in the uncoupled samples.

The Raman spectra of a set of samples with large twist angles (θ > 10°) exhibit no E-mode splitting; see Supplementary Figure S8. This indicates that the observed Raman splitting is an exclusive feature of the aligned samples (θ ≈ 0°). We note that the activation of the A_2u_ mode of MoSe_2_ remains unaffected by the twist angle and can be thus considered a fingerprint of the good coupling between the two monolayers. We verify that the E-mode splitting observed in MoS_2_/MoSe_2_ heterostructures is universal for other heterosystems with similar lattice mismatches; see Supplementary Figures S12 and S13 (Section S4).

**Figure 2:**
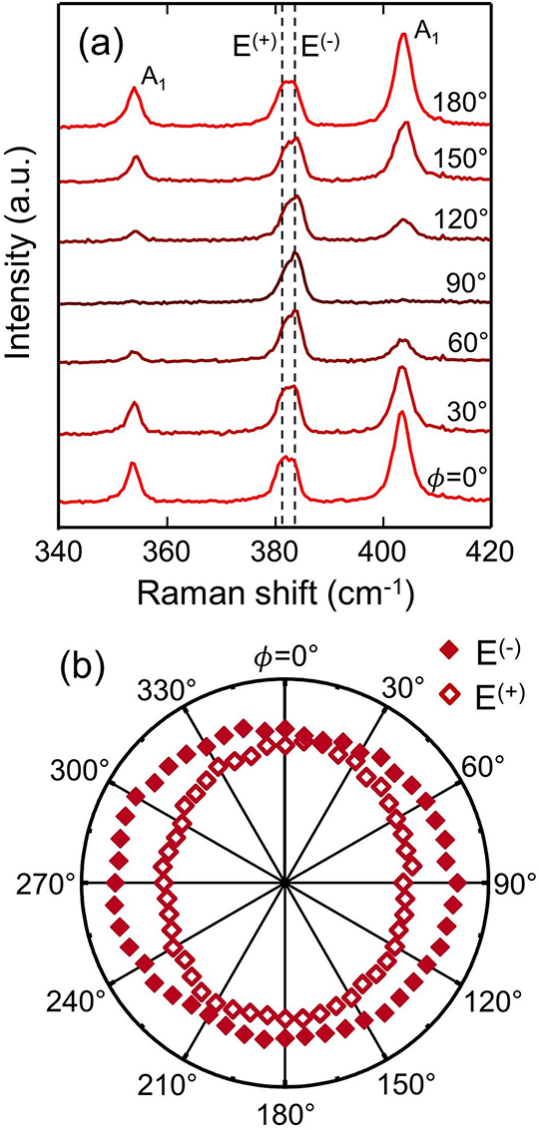
Linear-polarization dependence of the Raman spectroscopy of reconstructed MoS_2_/MoSe_2_ heterobilayers. (a) Polarized Raman spectra, where the angle of the detection polarizer, ϕ, was varied while keeping the excitation polarizer fixed. (b) Radial plot of E^(+)^ and E^(−)^ mode intensities. Note that the *C*_3*v*_ symmetry of the MoS_2_/MoSe_2_ heterobilayer further reduces to *C*_*s*_ under uniaxial strain, and therefore, the notation of the E^(+)^ and E^(−)^ Raman modes should be adjusted correspondingly, i.e., A′^(+)^ and A′^(−)^.^49^

To shed more light on the type and levels of deformation in heterostructures stacked at θ ≈ 0°, we map out the dependence of the Raman spectra on the angle of linearly polarized scattered light (ϕ); see Figure 2(a). As expected, the intensity of the out-of-plane A_1_ modes is highly dependent on the polarization angle, including the coupling-activated A_1_ mode at 354 cm^–1^.^54^ We find that the E mode is a combination of (at least) two modes, similar to the peak splitting observed in uniaxially strained MoS_2_.^48,49,54^ The intensities of the two peak components—centered at 381.5 and 383.5 cm^–1^—are displayed in the polar plot of Figure 2(b). The two components can be assigned to E^(−)^ and E^(+)^; they arise due to the symmetry breaking of the doubly degenerate E phonon under anisotropic deformation.^55^ The splitting and shift of the E mode—which is not accompanied by an apparent shift of the out-of-plane A mode—is characteristic of in-plane uniaxial strain in MoS_2_.^48^ However, in pure uniaxial strain experiments, the intensity of the E-mode components can be fully controlled by modifying the polarization angle.^54^ In our case, none of the peaks completely vanish at any polarization, thus evidencing that the strain is not purely uniaxial and that a more complex strain scenario develops in strongly interacting MoS_2_/MoSe_2_ heterosystems.

### Atomic Reconstruction of MoS_2_/MoSe_2_ Moiré Superlattices

**Figure 3:**
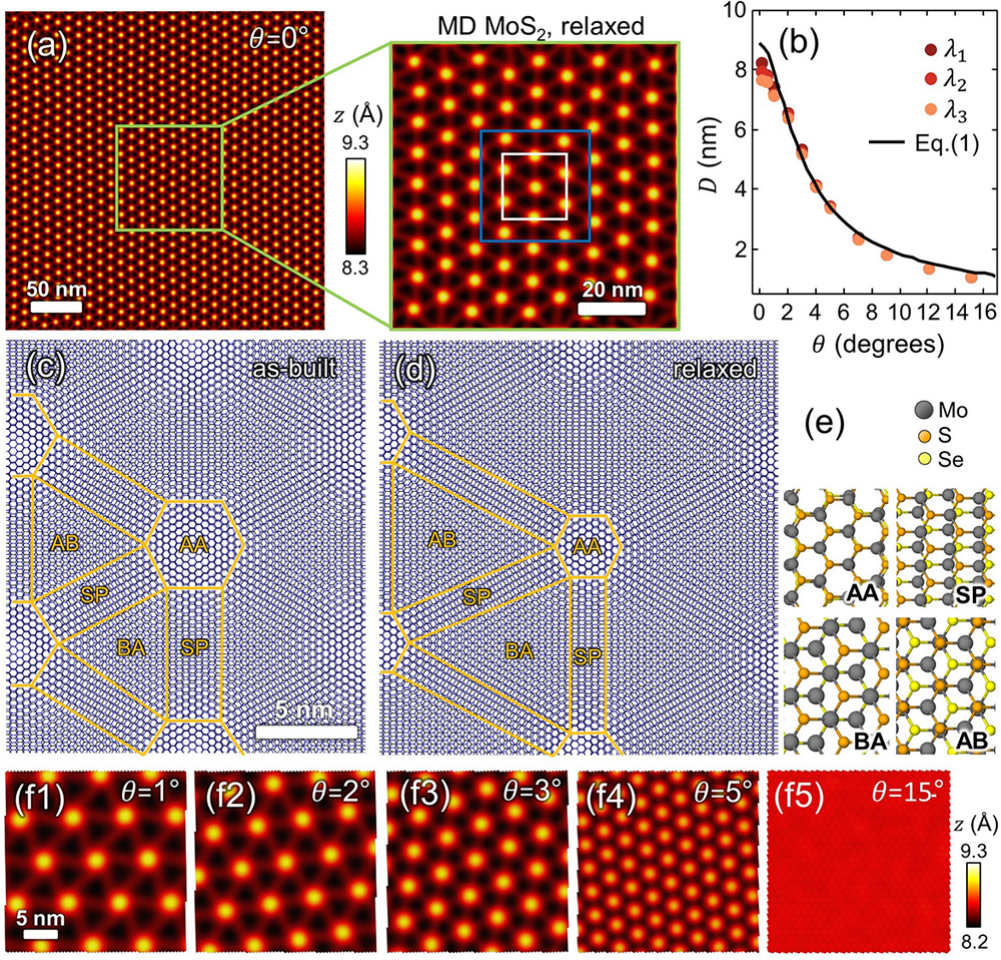
Lattice reconstruction in twisted MoS_2_/MoSe_2_ heterostructures. The MD topography maps of relaxed MoS_2_ membranes (θ = 0°) exhibit good scalability of the moiré patterns with areal dimensions decreasing from 240 × 240 nm^2^ (a) to 80 × 80 nm^2^ (inset). (b) Moiré periodicity, *D*, as a function of the twist angle, θ, obtained from the MD simulations. The data points correspond to the three lattice parameters (λ_1–3_) of the hexagonal moiré patterns captured via our FTT analysis. The representation of intralayer bonds (“wire model”, blue for MoS_2_, black for MoSe_2_) reveals the generation of specific stacking domains in the (c) as-built and (d) relaxed MoS_2_/MoSe_2_ configurations with θ = 0°. (e) The characteristic stacking configurations appearing in reconstructed MoS_2_/MoSe_2_ heterostructures. (f1–f5) Moiré patterns in nonprestrained MoS_2_ membranes with varying θ.

We perform MD simulations to analyze the topography of twisted MoS_2_/MoSe_2_/Au heterosystems with increasing θ (from 0 to 30°); see Computational Methods and Supplementary Section S5 for further details. With small twist angles (0 ≤ θ < 2°), our simulations reveal the systematic development of moiré superlattices characterized by a large periodicity (*D* ≈ 8 nm); cf. Figure 3(a). This figure shows the good scalability found in the moiré patterns obtained in our MD simulations when the area of the freestanding MoS_2_ membrane is decreased from 240 × 240 to 80 × 80 nm^2^.

The MD topography maps of the relaxed MoS_2_ membranes are analyzed via fast Fourier transform (FFT), which evidence the hexagonal superlattice of the moiré patterns in the reciprocal space. The moiré lattice constants (λ_1–3_) are then extracted from the radial profiles; see Supplementary Figure S15 (Section S6). Figure 3(b) provides the extracted superlattice constants as a function of θ. Overall, our analysis shows excellent agreement with the reciprocal-space model described by Chen et al.,^56^ which anticipates the values of *D* as a function of θ through the equation
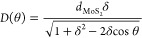
1Here, δ is the lattice-parameter mismatch between the MoSe_2_ and MoS_2_ crystals (δ = *d*_MoSe_2__/*d*_MoS_2__ = 1.0375, in reference to the *d*_MoSe_2__ and *d*_MoS_2__ values from the empirical potentials used in the MD simulations). However, for θ < 2°, we observe that the moiré lattice constants obtained from FFT slightly deviate from the model (eq 1) due to the pronounced lattice reconstruction occurring in MoS_2_/MoSe_2_ heterostructures. With θ ≤ 1° (i.e., near-0° conditions), the relaxed hexagonal patterns exhibit the largest anisotropy, as indicated by the marked fluctuations in the lattice constants extracted for the three directions of the hexagonal moiré patterns (Figure 3(f1)).

Our MD results provide a mechanistic rationale for the atomic reconstruction occurring in MoS_2_/MoSe_2_ heterobilayers. Figures 3(c,d) shows that the expansion of the AB and BA stacking domains plays a key role in the formation of moiré superlattices with low twist angles. Furthermore, such a domain expansion leads to the shrinkage of the AA stacking sites in the relaxed configuration. A scheme of the stacking configurations formed in MoS_2_/MoSe_2_ heterostructures is given in Figure 3(e). Similar structural reconstructions have been recently observed in WS_2_–WSe_2_ moiré heterostructures.^57^

With increasing θ values (2 < θ < 10°), the moiré periodicity in our relaxed MoS_2_ membranes drops as compared to the near-0° cases; see Figures 3(b,f2–f4). Lattice reconstruction, thus, is hindered with larger twist angles (θ > 10°), which produce moiré patterns characterized by a small periodicity (*D* < 2 nm; cf. Figure 3(b)) and mild out-of-plane deformations (Figure 3(f5)).

### Twist-Angle-Dependent Strains in MoS_2_/MoSe_2_ Heterostructures

The intralayer local strains, ε_local_, in our relaxed MD membranes are extracted from the components of the strain tensors defining the relaxation of the MoS_2_/MoSe_2_ heterostructure; see Supplementary Section S7 for further details on the procedure. By means of this analysis, we gauge the evolution of the local strain distributions with increasing θ (from 0 to 30°) in twisted MoS_2_/MoSe_2_/Au heterosystems. To eliminate potential edge effects, the local strain levels are systematically evaluated in a central 30 × 30 nm^2^ area of the relaxed MoS_2_ membranes (marked with a blue square in the inset to Figure 3(a)).

**Figure 4:**
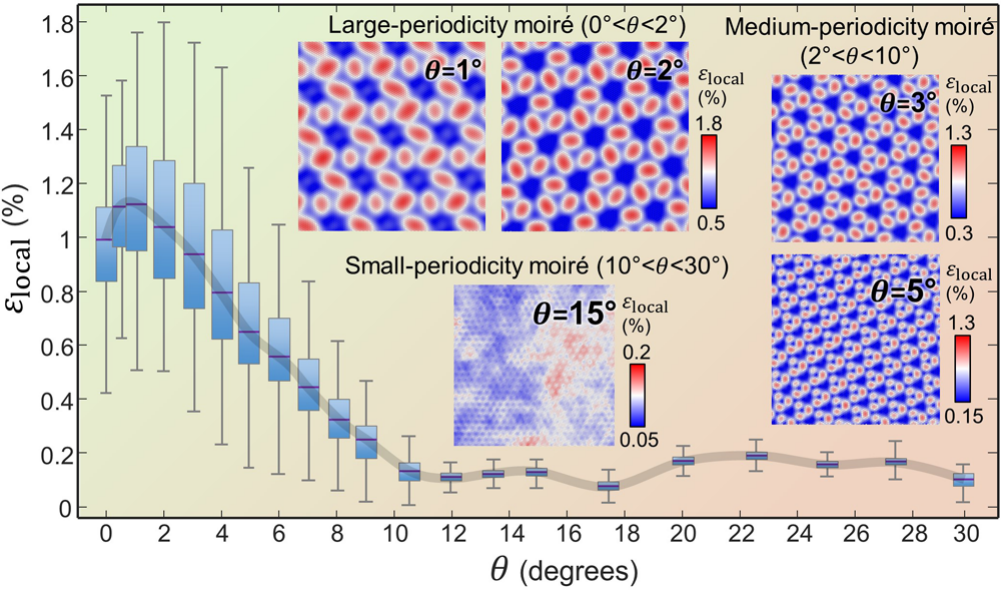
θ-dependent local strain in reconstructed (nonprestrained) MoS_2_/MoSe_2_ heterobilayers. The plot displays the box and whiskers representation of the ε_local_ data as a function of θ, where the violet line on each box indicates the median value, while the bottom and top edges indicate the 25th and 75th percentiles, respectively. The whiskers extend to the most extreme data points, not considering outliers (removed from the plot). The gray line marks the variation of the average ε_local_ in terms of the twist angle. The insets display the ε_local_ maps in MoS_2_ (central area of 30 × 30 nm^2^) with varying θ. The Supplementary Video shows the evolution of the strain patterns in the MoS_2_ with increasing twist angles.

The large-periodicity moiré superlattices formed with very small twist angles (0 ≤ θ < 2°) lead to an intricate distribution of local strains in our relaxed MoS_2_ membranes, where the largest ε_local_ levels are predominantly located at AA regions in the reconstructed heterostructures; see Supplementary Figure S16 (Section S7). As a result, complex heterostrain states are obtained (Supplementary Figure S17). Our results indicate that the attainment of such complex strain patterns is a fingerprint of lattice reconstruction in TMDC heterosystems with θ < 10°; cf. Figure 4. Although the largest moiré periodicity, *D*, in twisted MoS_2_/MoSe_2_ heterosystems is anticipated for θ = 0°,^56^ our ε_local_ calculations indicate that, at first, the strain levels gradually increase in MoS_2_ membranes with a twist angle that varies from θ = 0° to θ = 1°. We find that with θ = 1°, the MoS_2_/MoSe_2_ heterosystem produces the largest average ε_local_ value (∼1.15%). With increasing θ values (2 < θ < 10°), the average strain levels drop in our relaxed MoS_2_ membranes, as compared to the near-0° cases. The small-periodicity moiré patterns associated with larger twist angles (θ > 10°) produce low ε_local_ levels (with average values of ≈0.15%; cf. Figure 4).

**Figure 5:**
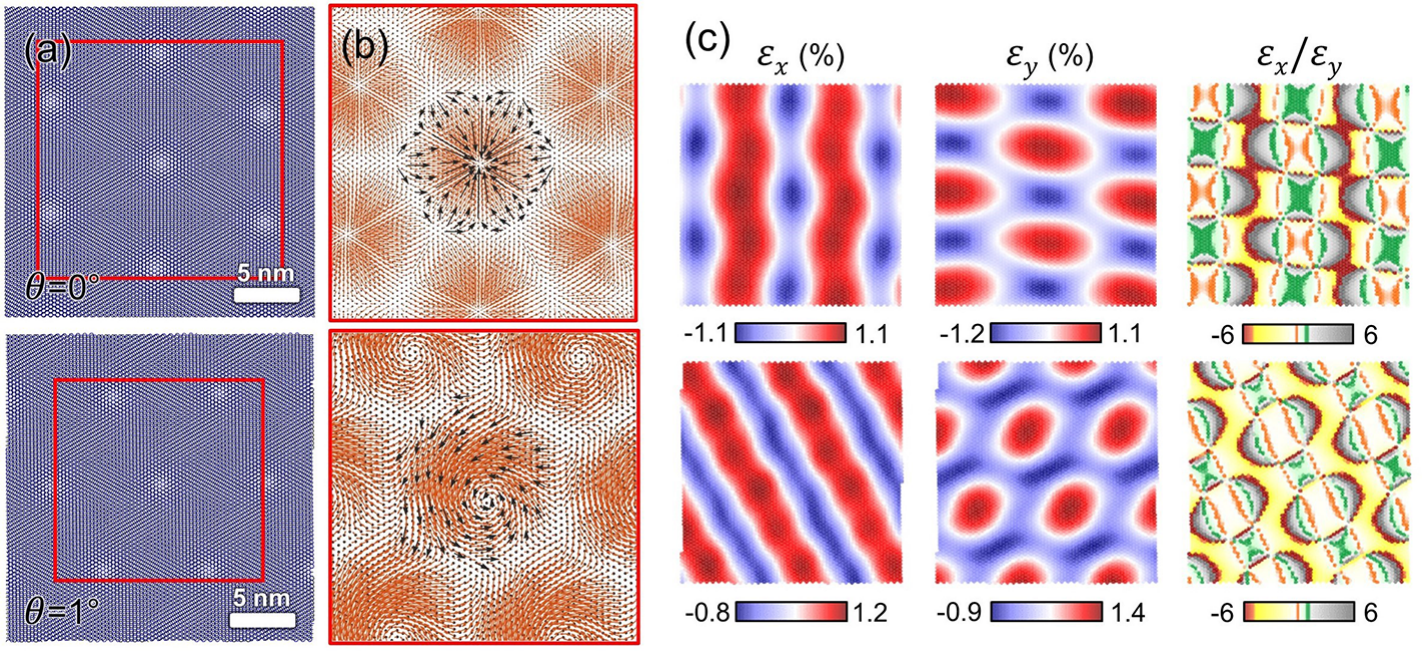
Displacement fields and strain states in reconstructed MoS_2_/MoSe_2_ heterobilayers. (a) Moiré patterns obtained in relaxed MoS_2_/MoSe_2_ heterosystems. (b) Vector fields of Mo displacements (inside the MoS_2_ area marked with a red square in (a)) during the MD relaxation runs. (c) Atomic-level strains in relaxed MoS_2_ membranes along the *x* (left) and *y* (middle) directions and their ratio (right). All plots in (a–c) correspond to 0° (top) and 1° (bottom) twist angles.

While the maps of local strain values from Figure 4 exhibit only minor anisotropy and heterogeneity in the (nonprestrained) MoS_2_ layers, a more revealing insight is provided by evaluating the displacement fields and the strain levels along defined directions. Figure 5(a–c) shows a detailed view of the moiré and strain patterns in the nonprestrained membranes under twist angles of θ = 0° (top row) and θ = 1° (bottom row) obtained from the MD simulations. Figure 5(a) displays the wire models of the intralayer bonds in both MoS_2_ and MoSe_2_ membranes, where the moiré patterns are visually manifested.

Figure 5(b) depicts the vector fields of the displacements of Mo atoms in the top (MoS_2_) layer upon lattice reconstruction. With θ = 0°, most of the Mo displacement vectors aim radially into the center of the AA sites. Only the displacement of the Mo atoms in between AA sites runs along the circumferential direction. However, with θ = 1°, the Mo displacements take on a direction that spirals toward the center of the AA site, resembling the “whirlpools” recently observed by Kim et al.^29^ in MoS_2_/WSe_2_ heterobilayers. Similar spiraling displacement patterns are observed with twist angles varying from 0.5 to 2°; see Supplementary Figure S18 (Section S8). These patterns gradually vanish with increasing θ. Under high twist angles (θ > 10°)—which hinder lattice reconstruction—an absence of such displacement fields in MoS_2_ is observed; see Supplementary Figure S19 (Section S8).

The distributions of the strain components in the *x* and *y* directions (ε_*x*_ and ε_*y*_, respectively) and their ratio (ε_*x*_/ε_*y*_) are shown in Figure 5(c). In the ε_*x*_/ε_*y*_ distributions, the green color corresponds to ε_*x*_/ε_*y*_ = 1 ± 0.1, that is, to regions with mostly biaxial strain. Biaxial states are predominantly located at the AA sites, but their contribution vanishes with increasing θ; compare the ε_*x*_/ε_*y*_ maps for θ = 0° and θ = 1° in Figure 5(c). Also, a localized strain distribution can be observed for approximately uniaxial strains (red hues) with – (ε_*x*_/ε_*y*_) = ν ± 0.1, where ν is the Poisson’s ratio set to 0.2 to cover the span of ν values (0.1–0.3) found in the literature.^58−60^ All other colors (yellows, grays) belong to areas where ε_*x*_/ε_*y*_ describes neither biaxial nor uniaxial straining states (i.e., shear strains).

### Effect of Prestraining in MoS_2_/MoSe_2_ Moiré Superlattices

**Figure 6:**
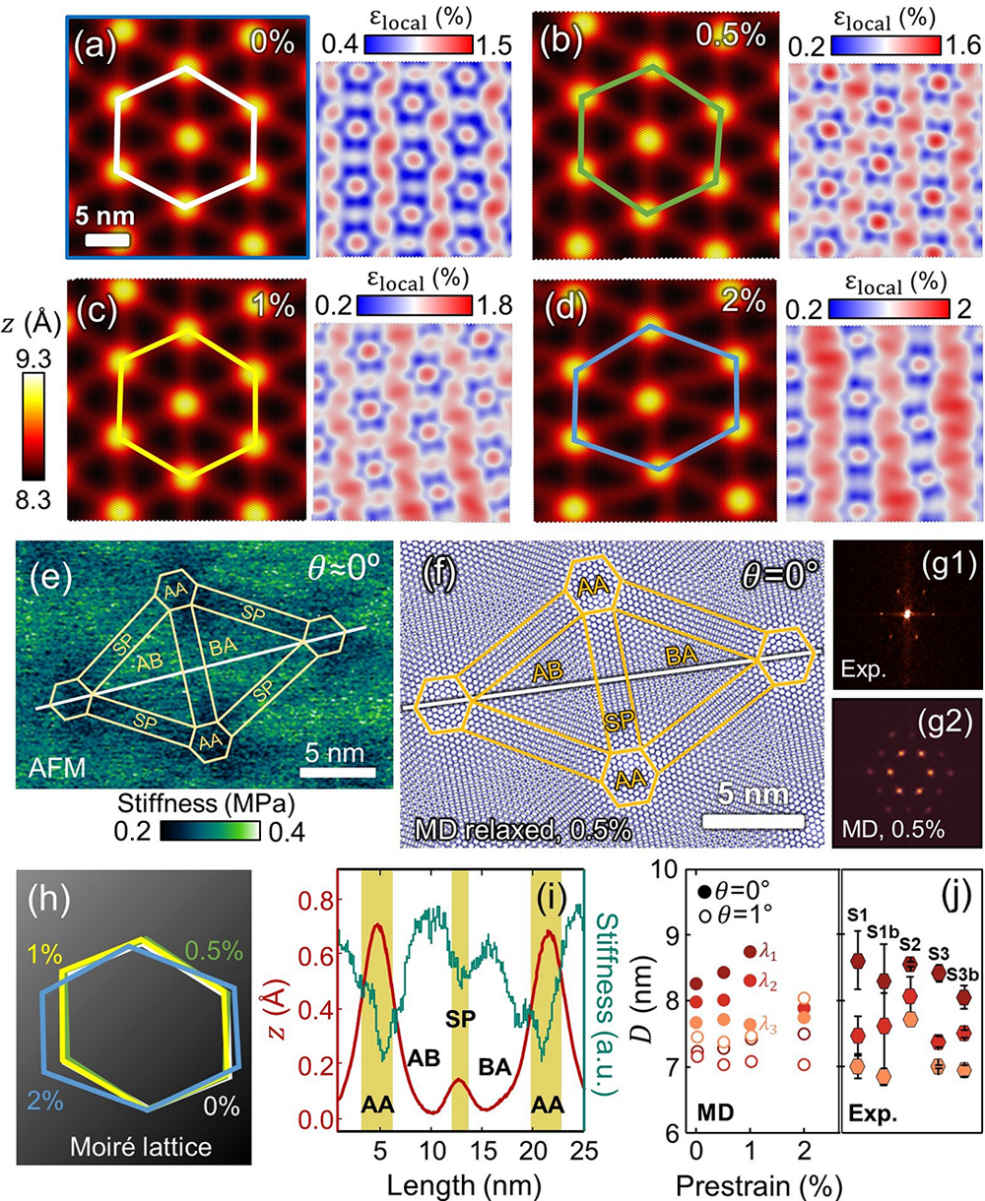
Effect of prestraining in reconstructed MoS_2_/MoSe_2_ heterobilayers with near-0° twist angle. (a–d) Moiré and strain patterns in relaxed MD MoS_2_ membranes (θ = 0°) with prestrain levels ranging from 0 to 2%. The topographies and ε_local_ maps are extracted from the central area of 30 × 30 nm^2^. (e) AFM stiffness image taken in Peak Force mode; see Supplementary Figure S9(b). (f) Stacking domains formed during lattice reconstruction in the uniaxially prestrained (0.5%) MD MoS_2_ membrane (θ = 0°). The 2D FFT images obtained from the AFM topography of Figure 1(c) and from MD topography of (b) are provided in (g1, g2), respectively. A schematic representation of the role of prestraining in the formation of relaxed moiré lattices is given in (h), where the hexagonal patterns correspond to those marked in (a–d). (i) AFM stiffness (green) and MoS_2_ MD height (red) profiles, marked with white lines in (e, f), respectively. (j) Moiré periodicities as a function of the prestrain level obtained from the MD simulations (left). Experimental λ values (right) are displayed for three distinct coupled MoS_2_/MoSe_2_ samples. Suffix b indicates a different area measured in sample S.

By MD, we also evaluate the role of uniaxial prestraining in the resulting topography and strain maps of MoS_2_/MoSe_2_ heterostructures with small twist angles, ranging from θ = 0° to θ = 2°. In our MD heterosystems, prestraining is introduced in the MoS_2_ along the armchair (*x*) direction. Figure 6(a–d) displays the evolution of the moiré patterns together with the ε_local_ maps with increasing prestrain levels under fixed θ = 0°. As expected, the ε_local_ distribution and moiré lattices become progressively more anisotropic with increasing prestraining. For comparison, the hexagonal moiré cells extracted from our relaxed MoS_2_ membranes with different levels of prestraining are brought together in Figure 6(h).

Figure 6(e) shows the experimental AFM-measured stiffness map of the coupled sample (displayed in Figure 1(c)), where the triangular-shaped AB (and BA) domains are clearly visible. For comparison, the resulting stacking domains found in the relaxed MD MoS_2_/MoSe_2_ heterosystem with a uniaxial prestraining of 0.5% and θ = 0° are shown in Figure 6(f). Note that the mechanisms of stacking-domain shrinkage/expansion in MoS_2_ also lead to the complex atomic displacement fields observed in our MD heterostructures with near-0° twist angles (Figure 5(b)). In this regard, the formation of large AB (and BA) domains is then limited to small twist angles. The AFM stiffness and MD topography profiles obtained from the linescans marked in Figure 6(e, f), respectively, are plotted together in Figure 6(i). Overall, these results reveal excellent agreement between the experiments and the MD simulations.

Our FFT analysis of the MD MoS_2_ topographies with twist angles θ = 0° and θ = 1° under different prestrain levels suggests the presence of three distinct values in the moiré lattice constants (λ_1–3_), thus revealing the onset of anisotropy in the moiré cells as a result of prestraining (comparison of the λ_1–3_ values from nonprestrained vs prestrained MoS_2_ is shown in Figures 3(b) and 6(j), respectively). The same FFT analysis was performed for the AFM topography images, where the λ_1–3_ were extracted under different scanning directions for several experimental samples; see Supplementary Figures S3 and S4 (Section S3). Averaging the corresponding λ_1–3_ values for a given sample area allows us to reduce the effect of measurement fluctuations and piezo distortions (indicated as error bars in Figure 6(j)). This analysis reveals that the experimental moiré patterns also provide three unequal superlattice constants ranging from ∼7 nm to ∼8.5 nm. Such a large anisotropy provides another indication that our exfoliated MoS_2_ membranes exhibit mild levels of prestraining (∼0.5%), which slightly vary between different samples and also between different areas in one sample.

To exclude possible AFM experimental artifacts, Supplementary Figures S10 and S11 (Section S3) display the outcome of measuring with parallel vs perpendicular scans and measuring with top-down vs bottom-up scans in the same sample area. The only negligible differences between the various scanning conditions confirm that the moiré lattice distortion and the λ_1–3_ values are real.

### Simulated Raman Shift Distribution of Reconstructed MoS_2_

The strain distributions calculated from MD simulations are converted into Raman shifts of the MoS_2_ E^(+)^ (ω_E_^+^) and E^(−)^ (ω_E_^–^) modes from the components of the strain tensor ε_*x*_, ε_*y*_, and ε_*xy*_ (extracted from a central area of 30 × 30 nm^2^ in the relaxed MoS_2_ membranes) according to^61^
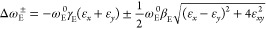
2where ω_E_^0^ is the E-mode frequency without strain (taken as 385.5 cm^–1^ as a relative reference to the 1L MoS_2_ on a SiO_2_/Si substrate; see the spectrum in Supplementary Figure S5 (Section S3)), γ_E_ is the Grüneisen parameter, and β_E_ is the shear-strain phonon deformation potential. We adopt γ_E_ = 1.1 and β_E_ = 0.78, which were obtained in a four-point bending (i.e., uniaxial) experiment conducted by Conley et al.^62^ The “Raman spectrum” is then obtained by calculating the frequencies of the E^(+)^ (ω_E_^+^) and E^(−)^ (ω_E_^–^) modes for every atom contained within the simulated area (of 30 × 30 nm^2^) and summing up all the contributions with the full-width-at-half-maximum (fwhm) set to 2 cm^–1^ for each calculated individual peak. It must be emphasized that the simplified simulations of the Raman shift merely capture the distribution of the strain-induced E-mode shifts. However, this allows for a comparative analysis of moiré superlattices with large periodicity values that would not be accessible using common *ab initio* calculations.

**Figure 7:**
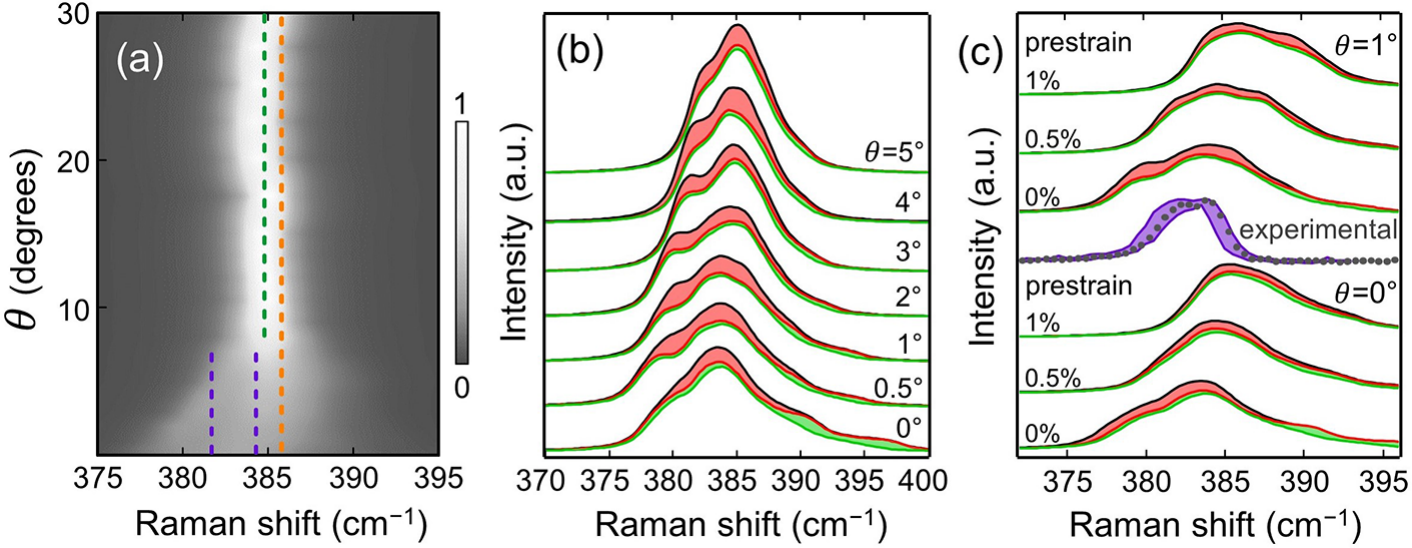
Simulated Raman spectra of reconstructed MoS_2_. (a) Distribution of local strains expressed in terms of the Raman shift of the MoS_2_ E mode according to eq 2 as a function of the twist angle, θ. The color scale corresponds to the normalized spectral intensity. The dashed lines in (a) indicate the experimentally obtained ω_E_^+^ and ω_E_^–^ (magenta), ω_E_ from the heterostructure with no visible moiré pattern (green), and ω_E_^0^ from bare MoS_2_ (red). Simulated Raman spectra of twisted MoS_2_ as a function of θ and the prestrain are given in (b, c), respectively. The red and green fills in (b, c) depict the contribution of pure uniaxial and biaxial strain states, respectively. For comparison, the E-mode experimental spectrum extracted from the polarization-dependent measurements (Figure 2(a)) is shown in (b), where the dots represent the average, and the magenta fills the minimum–maximum range.

The overall evolution of the simulated spectra of nonprestrained MoS_2_ is plotted in Figure 7(a) for twist angles ranging from 0 to 30°. For twist angles >10°, the E mode is symmetrical. The fwhm reaches down 2.2 cm^–1^, and the average value of ω_E_ is 384.8 ± 0.2 cm^–1^ (indicated by the green dashed line in Figure 7(a)), which exactly matches the measured ω_E_ of the noninteracting MoS_2_ depicted in Figure 1(a). For comparison, the red dashed line in Figure 7(a) displays the ω_E_ of the pristine MoS_2_ (at 385.5 cm^–1^). For θ < 10°, the strain distribution in the Raman spectra takes on a markedly asymmetric shape, with two (or more) components clearly present.

In the nonprestrained MoS_2_, the higher-frequency component is always more intense than the lower-frequency one (Figure 7(b)), thereby strongly resembling the experimental Raman spectra. For the prestrained MoS_2_, the E-mode components evolve in a more complicated manner. However, they still maintain the asymmetric shape for low twist angles; see Figure 7(b). For comparison, the experimental spectra (obtained from the E-mode region in Figure 2(a)) are included in Figure 7(c) where the magenta-filled area outlines the range in which the spectral intensity reaches the given pixel for all analyzer angles and the gray dots represent their average. The positions of the two experimental E-mode maxima are also displayed in Figure 7(a) with magenta dashed lines. The net biaxial and uniaxial contributions to the simulated Raman spectra are marked in Figures 7(b, c) by green and red colors, respectively. The remaining areas under the curves (white) come from Raman shifts where ε_*x*_/ε_*y*_ corresponds to various shear strains. More importantly, the spectrum areas filled by uniaxial states in the simulated Raman correlate with the changes in the experimental spectrum observed upon changing the analyzer angle; compare in Figure 7(c) the red areas (simulated spectra) with the magenta area (experimental spectrum). The agreement found between the simulated and measured strain distributions allows us to roughly estimate that the interrogated bilayer system has indeed a low twist angle (near 0°), as indicated by the E-mode splitting from Figure 7(c). Moreover, the resulting Raman shift indicates that our exfoliated MoS_2_ membranes could be slightly prestrained.

We note, however, that the accuracy of the Raman-spectrum simulation may be hampered by the uncertainty in the parameters entering eq 2. There is a large span of γ_E_ values in the literature from biaxial deformation tests, reaching down 0.60–0.64.^63,64^ Even though we adopted the values reported by Conley et al.^62^ for our calculations of the Raman shifts to maintain a single source for both parameters, the use of a significantly lower value for the Grüneisen parameter (i.e., γ_E_ = 0.60) can change the outcome by shifting the E mode (∼1.5 cm^–1^) to higher wavenumbers and by narrowing the distribution (∼1 cm^–1^); see Supplementary Figure S20 (Section S9). The comparison between the experiments and the simulations is thus only tentative but it corroborates the observations made in the previous sections.

## Conclusions

In this work, we evaluate local strains in reconstructed MoS_2_/MoSe_2_ moiré superlattices. Our study combines AFM and Raman measurements with MD simulations. We find that MoS_2_/MoSe_2_ heterostructures with twist angles between 0 and 2° exhibit the highest levels of local strain due to specific structural reconstructions of the stacking domains that occur in the MoS_2_. Lattice reconstruction is hindered for heterostructures with larger twist angles (>10°). The MD simulations indicate that the formation of moiré patterns in TMDC heterostructures leads to a complex distribution of local strains with a combined deformation state of uniaxial, biaxial, and shear components. These findings are supported by our polarization-dependent Raman spectroscopy measurements. The twist-angle-dependent lattice reconstruction and highly localized strain distribution are in line with the previously reported observations of θ-dependent emission energy of the interlayer excitons^43,44^ and confirm the potential for manipulating moiré quantum wells simply by proper oriented stacking. Furthermore, anisotropy of the moiré superlattice may result from the introduction of heterostrain during the stacking process. The experiments and simulations suggest that moiré anisotropy can be tuned by controlling the uniaxial prestraining of the top layer, thereby enabling the development of TMDC moiré optoelectronic devices with tailored directional properties.

## Experimental Methodology

### Sample Fabrication

Our MoS_2_/MoSe_2_ heterostructures were prepared by mechanical exfoliation of bulk crystals (HQ graphene). The MoS_2_ and MoSe_2_ monolayers were separately exfoliated on polydimethylsiloxane stamps (Gelfilm by Gelpak) and sequentially transferred to SiO_2_/Si substrates (300 nm SiO_2_ thickness) using a dry-transfer technique.^65^ Exfoliated monolayers were identified using PL spectroscopy.

### Optical and Surface Characterization

Raman and PL measurements were performed in a LabRAM HR Evolution spectrometer (Horiba Scientific) with 532 nm laser excitation using a 100× objective (0.8 NA). Diffraction gratings of 1800 and 150 l/mm, giving pixel-to-pixel resolutions of 0.5 and 7.0 cm^–1^, were used for the Raman and PL characterizations, respectively. For all measurements, the laser power was set between 40 and 80 μW. The angle of the linear polarized incident light was fixed and the angle of the scattered light was selected by rotating the analyzer.

AFM topography and stiffness images were taken with a Bruker Dimension ICON in PeakForce Tapping mode using Scanasyst Air probes (Bruker Corp.).

## Computational Methods

We perform large-scale MD simulations of MoS_2_/MoSe_2_/Au heterosystems employing the LAMMPS code.^66^ The details of the construction of the all-atom heterosystems are given in Supplementary Section S5 and Figure S14. The Stillinger-Weber (SW) potential is employed to describe the intralayer atomic interactions in the MoS_2_ and MoSe_2_ membranes. We adopt the SW parameters obtained by Kandemir et al.,^67^ which generate consistent results with density functional theory.

The constituent layers (the top MoS_2_ membrane, the intermediate MoSe_2_ membrane, and the bottom Au substrate) are mutually adhered through weak-bond van der Waals (vdW) forces. To model these interactions, the archetypal 12–6 Lennard-Jones (LJ) potential—Supplementary eq (S4) (Section S10)—is used to describe the vdW-type adhesion between the MoSe_2_ layer and the substrate. The values of the LJ parameters for the Mo–Au and Se–Au interactions are given in Supplementary Table S2 (Section S10). The LJ cutoff distance is set to 8.5 Å.

In addition, to model MoS_2_–MoSe_2_ interlayer forces, we employ the Kolmogorov-Crespi (KC) potential^68^ parametrized for TMDCs by Naik et al.^69^ This model incorporates a stacking-dependent term that enables interlayer sliding processes to generate different stacking configurations with different binding energies. The development of moiré patterns in 2D materials is fundamentally affected by this stacking dependence of the binding energy, which in turn cannot be captured by the simpler LJ model.^70^ We adopt the ẑ-normals simplification (KC-z),^69^ and we set a KC cutoff radius of 14 Å. An assessment on the formation of stacking domains *vis-á-vis* the employed interlayer (LJ vs KC) potentials is provided in Supplementary Sections S11 and S12 and Figures S22–S24.

The Au substrate is composed of two rigid (111)-oriented atomic layers. The interactions among the Au atoms are excluded from the calculations, and their positions remain fixed during the MD runs. Periodic boundary conditions are imposed on both sides of the computational domains, while the top and bottom walls are nonperiodic and fixed. The vertical dimension of the MD cells is set to 40 Å.

The MoS_2_/MoSe_2_/Au heterostructures are relaxed through a 1 ns run where the dynamics of the atoms in the MoS_2_/MoSe_2_ bilayer system follow the canonical *NVT* ensemble. We use a Nosé–Hoover (NH) thermostat with 3 NH chains^71^ to control the temperature of the membranes at 300 K. The computational time step is set to 1 fs. Atomic visualization and data analysis of the MD results are carried out with the OVITO package.^72^

## Supporting Information Available

Raman and AFM characterizations of additional MoS_2_/MoSe_2_ and MoS_2_/WSe_2_ samples. MD simulation details and effects of various simulation parameters on the coupling, strain, atomic displacements, and distribution of Raman shifts (PDF)
Supplementary video showing the evolution of local strain with increasing twist angle (MP4)
