# Supplementary material for: Complex Strain Scapes in Reconstructed Transition-Metal Dichalcogenide Moiré Superlattices
Source: ACS Nano. 2023 Apr 6;17(8):7787–96. doi: 10.1021/acsnano.3c00609 (PMC10134736; doi:10.1021/acsnano.3c00609)
Supplement: Supplementary file 1 — nn3c00609_si_001.pdf [file nn3c00609_si_001.pdf]

Supplementary information to the manuscript "*Complex Strain Scapes in Reconstructed Transition-Metal Dichalcogenide Moiré Superlattices*"

Álvaro Rodríguez [1,2,*], Javier Varillas [1,3,*], Golam Haider [1], Martin Kalbáč [1], and Otakar Frank [1,*]

[1] J. Heyrovsky Institute of Physical Chemistry, Czech Academy of Sciences, Dolejškova 2155/3, 18223 Prague 8, Czech Republic

[2] Materials Science Factory, Instituto de Ciencia de Materiales de Madrid, Consejo Superior de Investigaciones Científicas, 28049 Madrid, Spain

[3] Institute of Thermomechanics, Czech Academy of Sciences, Dolejškova 1402/5, 18200 Prague 8, Czech Republic

***E-mail addresses**: alvaro.rodriguez@csic.es; jvarillas@it.cas.cz; otakar.frank@jh-inst.cas.cz

S1. Moiré periodicities in TMDC homo- vs. heterobilayers

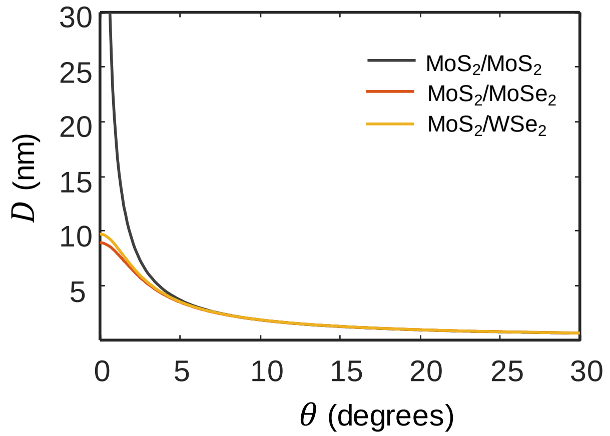

Figure S1. Moiré periodicity, D, as a function of the twist angle, θ, in MoS_2/MoS_2, $MoS_2/MoSe_2$, and MoS_2/WSe_2 bilayers, as obtained using Eq. (1) from the main text.

S2. Annealing effect on the interlayer spacing of $MoS_2/MoSe_2$ heterobilayers

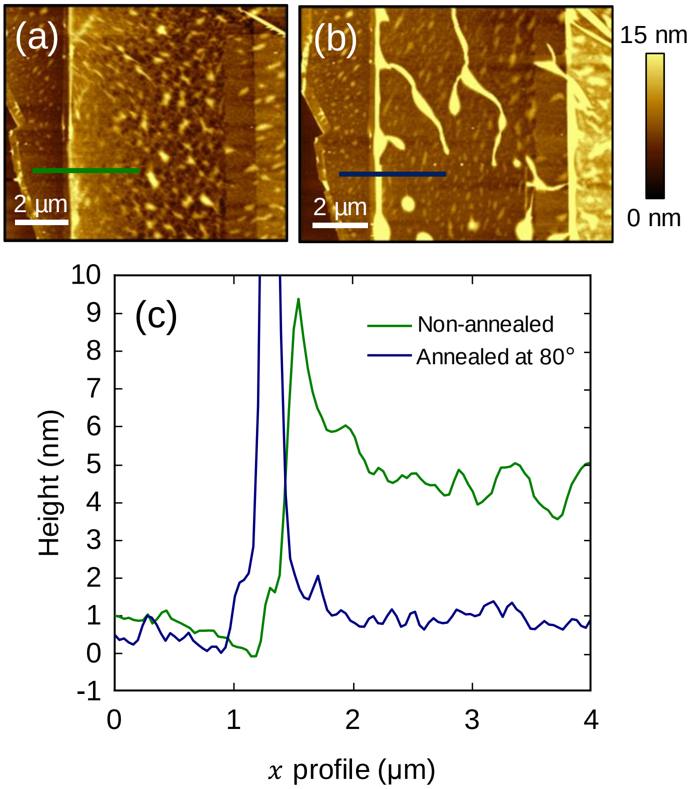

Figure S2. Topography images of a $MoS_2/MoSe_2$ heterobilayer (a) as-transferred and (b) after annealing at 80ºC for 10 min. (c) Height profiles of the interlayer spacing for the two cases.

S3. Moiré patterns in MoS_2/$MoSe_2$ bilayers

We fabricated five (5) MoS_2/$MoSe_2$ heterobilayer samples with a twist angle, θ, near 0º by the all-dry transfer method. In all cases, MoS_2 is the top layer in the stack. Figures S3(a, b) shows the optical image of the parallelly aligned heterobilayer along with an AFM topography snapshot of each sample. A moiré pattern with a periodicity $D \approx 8$ nm is observed for all the samples, confirming a twist angle of ≈0º. Larger values of θ would lead to periodicities smaller than 8 nm; see Fig. 3(b) in the main text. The FFT analyses of the superlattice constants (λ_{1-3}) (Figs. S3 and S4) reveal a certain degree of anisotropy in the moiré patterns evidenced by the dissimilar values of the three constants; cf. Fig. S3(c). We attribute this to the heterostrains caused during the stacking of the two monolayers. For further details, see the discussion given in the main text.

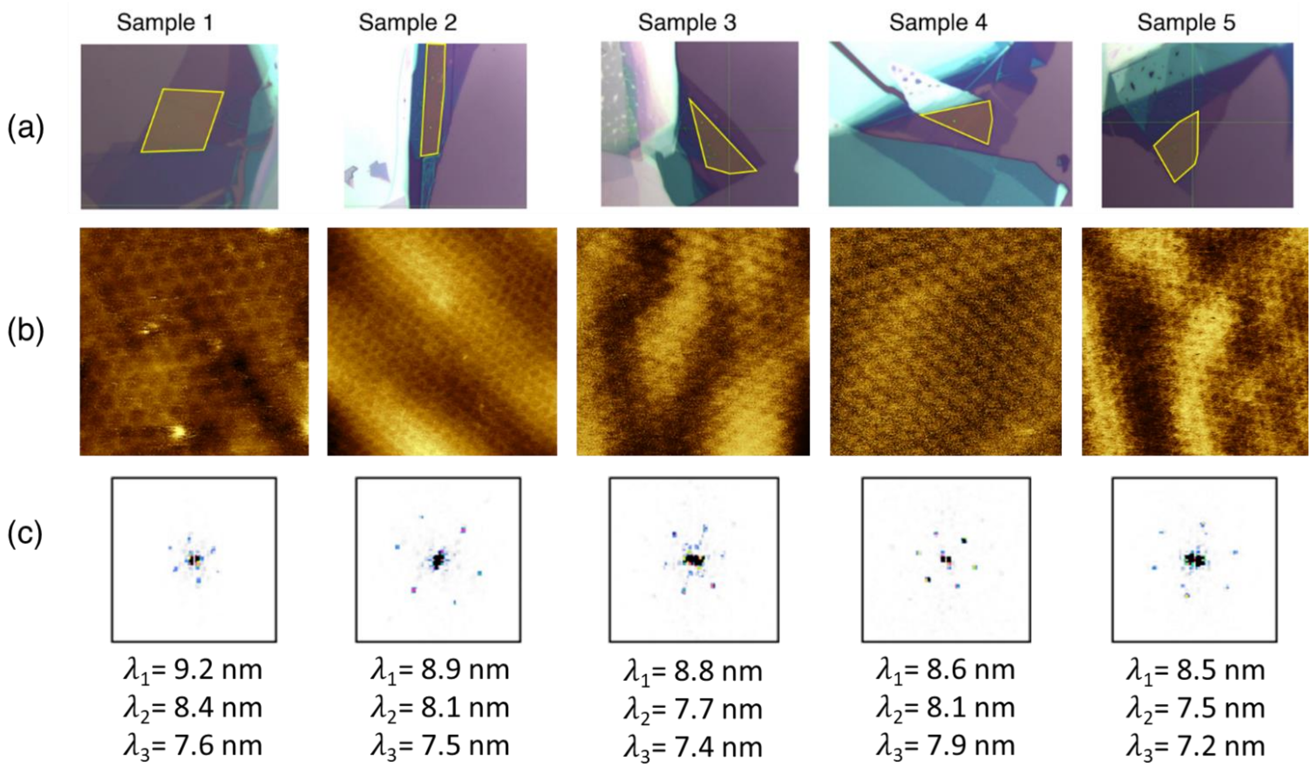

Figure S3. (a) Optical micrographs of five MoS_2/$MoSe_2$ bilayers. (b) Moiré patterns of the samples measured by AFM. (c) FFT analyses for each AFM topography image.

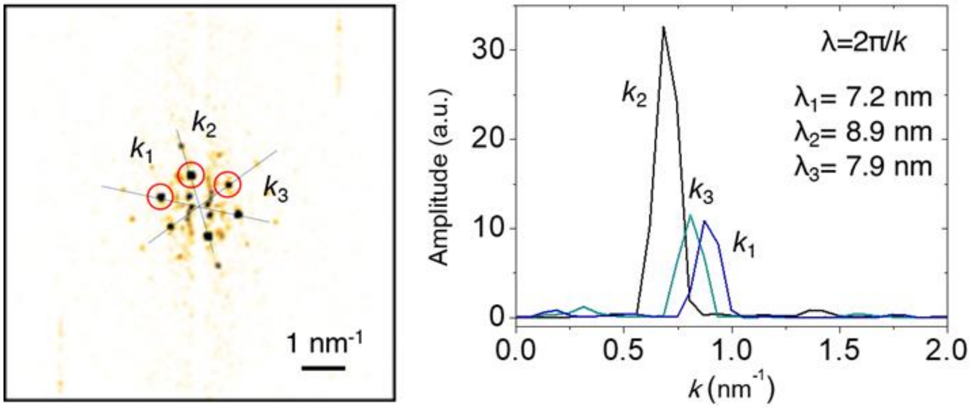

Figure S4. FFT analysis of moiré pattern obtained in the AFM topography image. The three superlattice constants are obtained from the reciprocal space.

The Raman spectra of the bilayer samples are contained in Fig. S5. The two distinctive features of coupled $MoS_2/MoSe_2$ heterobilayers are (i) the splitting of the E_{2g} mode ($\approx$ 385 cm^{-1} for 1L MoS_2) due to local strains induced by the atomic reconstruction of MoS_2, and (ii) the emergence of the A_{2u} mode of $MoSe_2$ at $\approx$ 354 cm^{-1}.

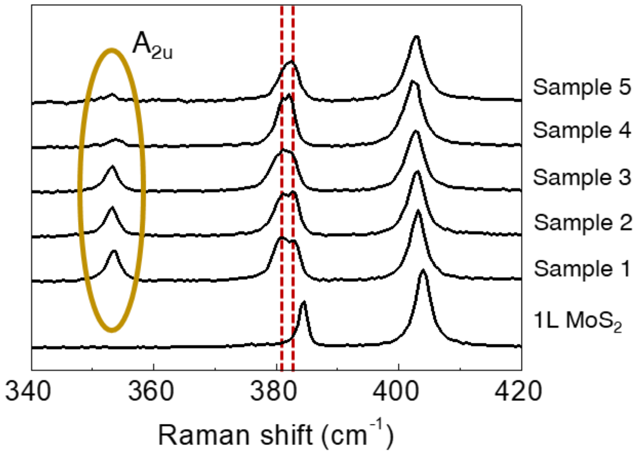

Figure S5. Raman spectra of $MoS_2/MoSe_2$ bilayers at $\theta \approx 0°$. The Raman spectrum of monolayer (1L) MoS_2 is shown for comparison.

A more detailed study of the Raman spectra is shown in Fig. S6(a). The Raman spectrum of $MoS_2/MoSe_2$ bilayer exhibits some weak Raman signals that become optically active in the bilayer as compared to the monolayer MoS_2. The correct notation of the Raman modes for

heterobilayers belonging to the C_{3v} point group should be E and A_1 for the in-plane and out-of-plane phonons, respectively. The equivalent modes for the homobilayers are shown in Fig.S6(c). Besides the abovementioned A_1 mode, other in-plane and out-of-plane modes are detected, which correspond to the E_{1g} and A_{2u} modes observed in the homobilayers, which are forbidden in the monolayers.

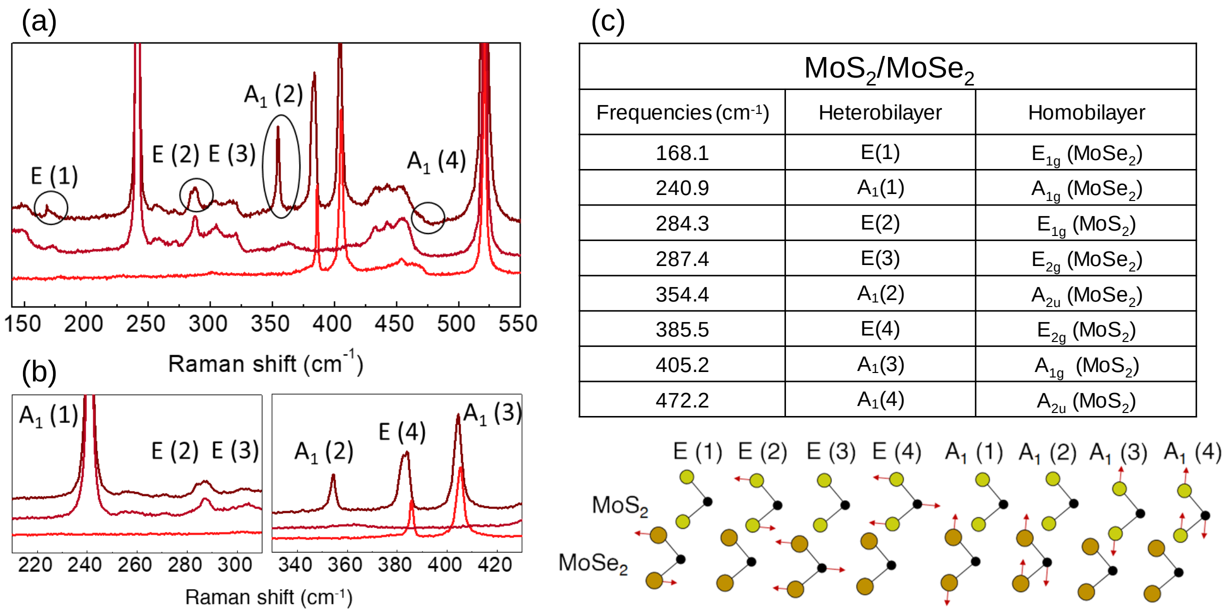

MoS$_2$/MoSe$_2$		
Frequencies (cm^{-1})	Heterobilayer	Homobilayer
168.1	E(1)	E_{1g} (MoSe$_2$)
240.9	A_1(1)	A_{1g} (MoSe$_2$)
284.3	E(2)	E_{1g} (MoS$_2$)
287.4	E(3)	E_{2g} (MoSe$_2$)
354.4	A_1(2)	A_{2u} (MoSe$_2$)
385.5	E(4)	E_{2g} (MoS$_2$)
405.2	A_1(3)	A_{1g} (MoS$_2$)
472.2	A_1(4)	A_{2u} (MoS$_2$)

Figure S6. Raman spectra of MoS$_2$/MoSe$_2$ bilayers with $\theta \approx 0°$. (a) Raman spectra of MoS$_2$ and MoSe$_2$ monolayers are shown for comparison. (b) Enlarged Raman spectrum of the region of interest showing the Raman modes active in the bilayer. (c) Raman modes of MoS$_2$/MoSe$_2$ heterobilayers.

To illustrate the homogeneity of the moiré pattern throughout the samples, we measured a large-area AFM topography scan of a heterobilayer. Figure S7(a) shows a uniform moiré pattern across the measured area, suggesting that bubbles are formed in the bottom layer. Figure S7(b) gives clear evidence of that the moiré patterns are only visible in the bilayer area (left-hand side of this figure), where the monolayer/bilayer edge is marked in yellow.

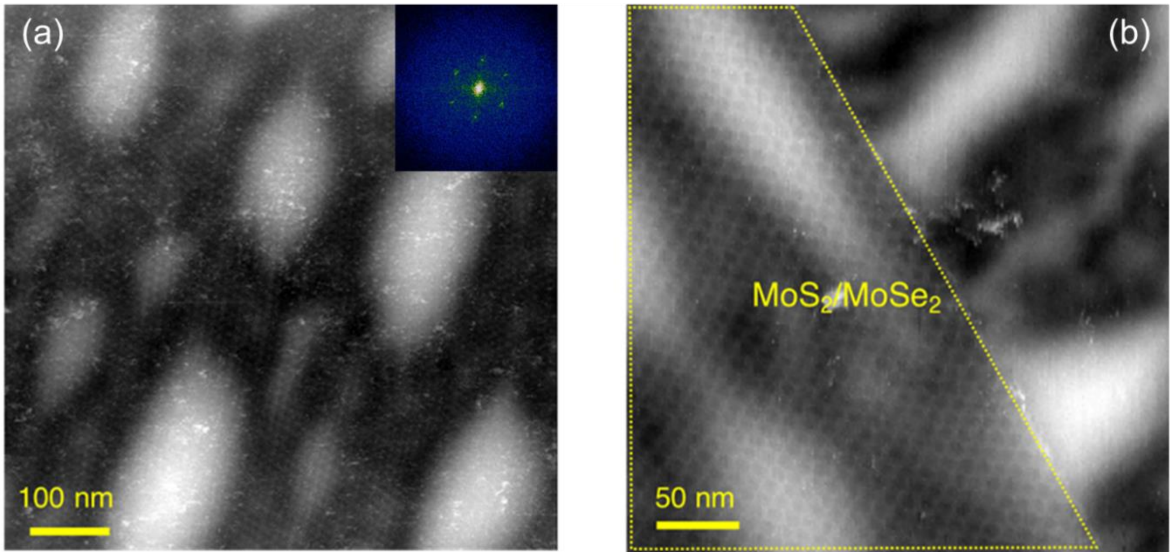

Figure S7. Large-area AFM topographies of a $MoS_2/MoSe_2$ heterobilayer with $\theta \approx 0°$. The inset in (a) show the FFT image of the moiré patterns in this area.

A set of heterobilayers with twist angle >10° was also fabricated in order to compare the resulting Raman features with those from the aligned (near-0° twist angle) heterobilayers. The Raman spectra of non-aligned ($\theta > 10°$) bilayers is shown in Fig. S8, where the absence of E_{2g} splitting is appreciable. This is consistent with the lack of lattice reconstruction found in our MD simulations of twisted $MoS_2/MoSe_2$ bilayers with $\theta > 10°$. The presence of the A_{2u} mode indicates the formation (or coupling) of the heterobilayer because this mode is forbidden in monolayer $MoSe_2$.

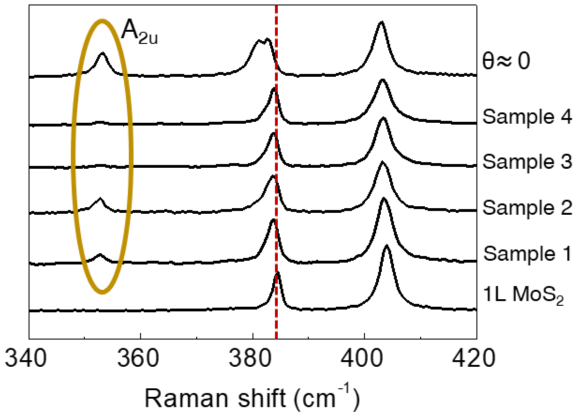

Figure S8. Raman spectra of MoS2/MoSe2 bilayers at $\theta > 10°$. For comparison, the spectrum of a bilayer with $\theta \approx 0°$ is plotted at the top of the figure, which shows a strong signal of the A_{2u} mode.

Although the moiré pattern is visible in the topography channel, the triangular domains of AB and BA stacking (see Figs.6(e) in the main text) become more evident in the stiffness channel, as observed in Fig. S9. Both topography and stiffness images are obtained from the same area. The Stiffness images were measured in Peak Force Tapping [1].

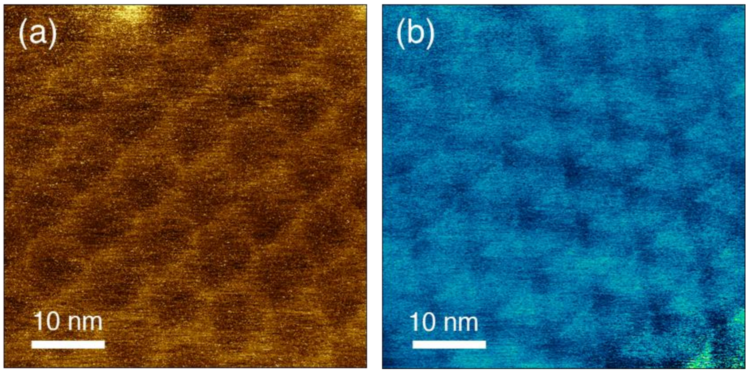

Figure S9. AFM topography and stiffness images of the same sample area ($\theta \approx 0°$) are given in (a) and (b), respectively.

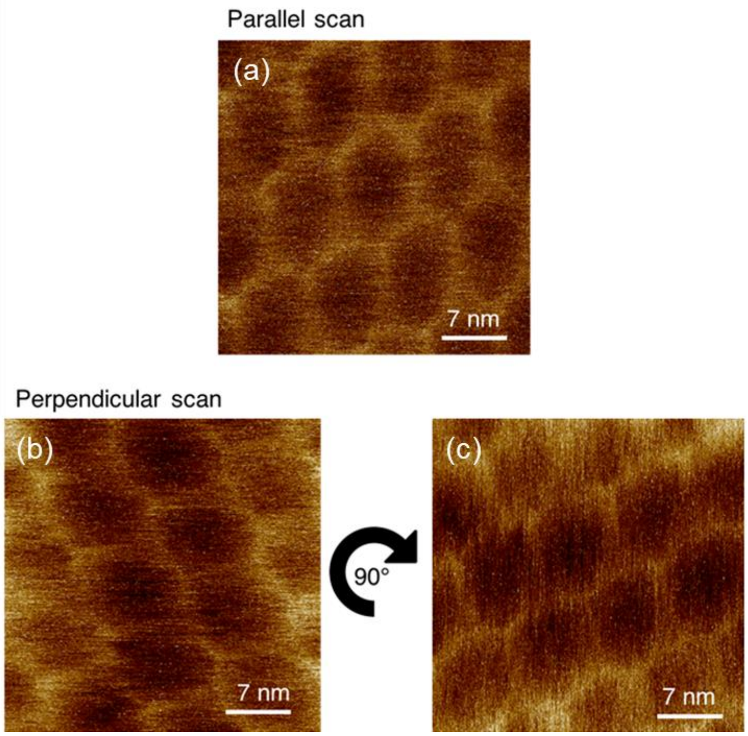

Figure S10. Deformation test of the piezo scanner in the x direction. (a) Scan parallel to the sample. Scans perpendicular to the sample of the same area are shown in (b, c). When rotating 90º the image, a similar deformation is observed. The moiré pattern distortion is then unaffected by the piezo scan direction, which indicates that moiré anisotropy is found in the sample.

Figure S10 illustrates the effect of the piezo deformation when scanning small areas. When scanning large areas with AFM, this effect is typically reduced. In our small-scan-area measurements, the moiré pattern appears to be deformed in the direction perpendicular to the scan, as indicated in Fig. S10(a). Then, the image taken at a 90° scan angle (Fig. S10(b)) reveals that the resulting distortion is parallel to the scan direction, thus ruling out the assumption that small scan areas may be causing the observed distortion. This indicates that an anisotropic moiré pattern is formed in the superlattices. In addition, Fig. S11 shows the difference of the AFM and FFT images taken when scanning from the top to the bottom and from the bottom to the top.

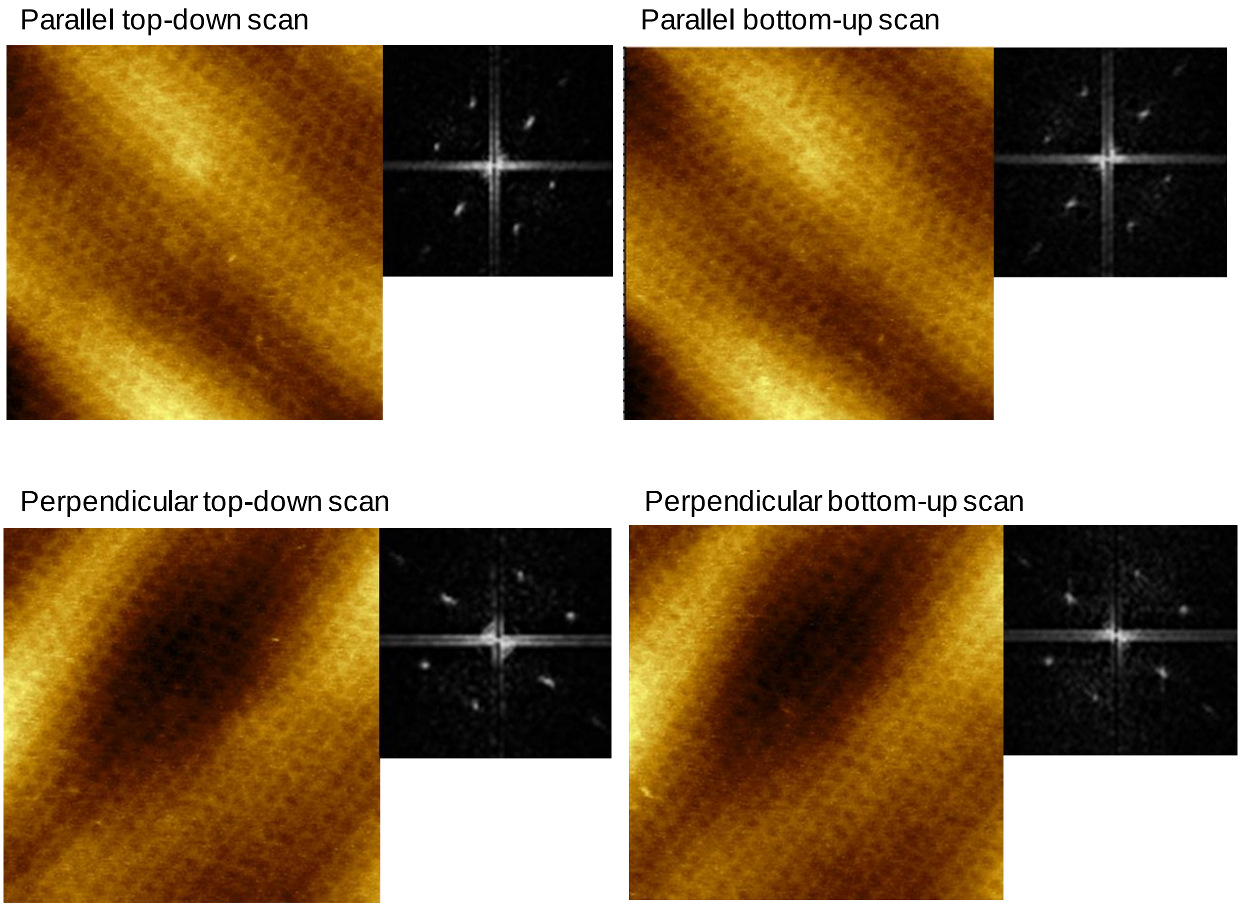

Figure S11. Large-scan test of the piezo scanner in the x direction. Similar deformations are observed for both scan directions, as observed in the insets showing the FFT analysis corresponding to each AFM image.

S4. Strain in MoS_2/WSe_2 superlattices

The Raman and PL spectra of a MoS_2/WSe_2 heterobilayer (prepared in the same way as the MoS_2/$MoSe_2$ heterobilayers) are shown in Fig. S12. The optical fingerprints resemble the case of MoS_2/$MoSe_2$ because of the emergence of new modes that can be assigned to the bilayer formation and the broadening of the E mode; compare Fig. S5 with Fig. S13.

The good interaction is supported by a strong interlayer exciton band at 1 eV that can be observed in the PL spectrum (Fig. S12(b)), in agreement with previous reports [2].

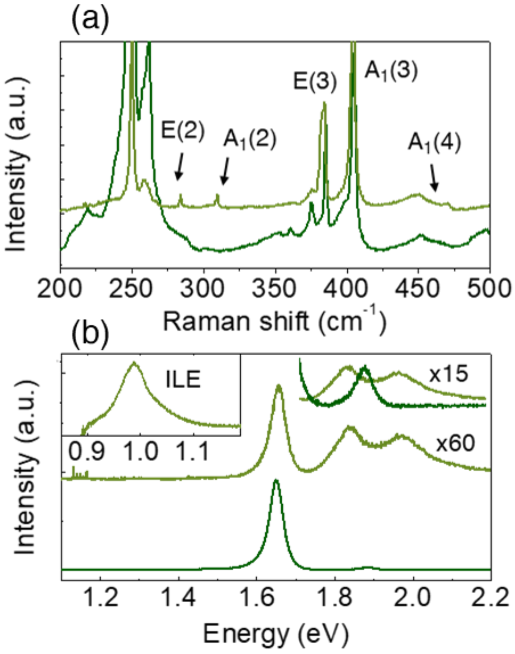

(c)

MoS_2/WSe_2		
Frequencies (cm^{-1})	Heterobilayer	Homobilayer
249.5	E(1)	E^1_{2g} (WSe_2)
249.5	A_1(1)	A_{1g} (WSe_2)
284.2	E(2)	E_{1g} (MoS_2)
309.5	A_1(2)	A_{2u} (WSe_2)
385.4	E(3)	E^1_{2g} (MoS_2)
404.9	A_1(3)	A_{1g} (MoS_2)
470.4	A_1(4)	A_{2u} (MoS_2)

Figure S12. (a) Raman and (b) PL spectra of coupled (light green) and uncoupled (dark green) MoS_2/WSe_2 heterobilayers with a twist angle of ≈ 0°. The characteristic Raman modes for a bilayer formation are appreciable in conjunction with the splitting/broadening of the MoS_2 E(3) mode. The inset to (b) shows the interlayer exciton at 1 eV, measured with an InGaAs detector. (c) Raman modes of MoS_2/WSe_2 heterobilayers.

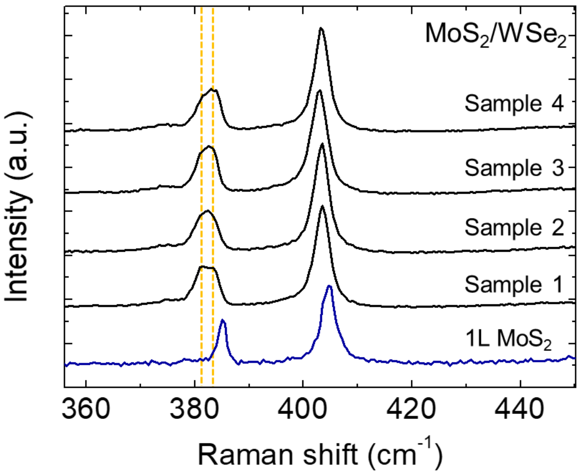

Figure S13. Raman spectra of several MoS_2/WSe_2 heterobilayer at $\theta \approx 0°$. Monolayer MoS_2 is shown for comparison. Broadening of the E mode can be observed in all the samples, similar as compared to the case of $MoS_2/MoSe_2$.

S5. The MD domain containing the $MoS_2/MoSe_2/Au$ heterosystem

We construct the $MoS_2/MoSe_2$ heterobilayer system by placing a freestanding MoS_2 membrane on top of a periodic $MoSe_2$ membrane, with respective sizes of l_{top} and l_{bot}, where $l_{\mathrm{top}} < l_{\mathrm{bot}}$. The x-direction follows the armchair orientation in both membranes. Additionally, we assess the effect of prestraining in a set of $MoS_2/MoSe_2$ systems. An initial tensile strain is homogeneously introduced in the top MoS_2 membrane, where the prestrain levels vary from 0.5% to 5%. Then, we impose a twist to the MoS_2 layer, where the vertical rotation axis crosses the Cartesian point $P = (l_{\mathrm{top}}/2, l_{\mathrm{bot}}/2)$. The rotation direction follows the right-hand rule; that is, for a positive twist angle, θ, the rotation is then anticlockwise on the x-y plane; see Fig. S14(a). The heterobilayer systems are built under three different sizes: (i) 80/100 nm ($l_{\mathrm{top}}/l_{\mathrm{bot}}$) for twist angles varying from 0º to 13.5º, (ii) 80/120 nm for large twist angles (from 15º to 30º), and (iii) 240/260 nm only for low twist angles (0º and 1º).

The $MoSe_2$ membrane is placed over a flat, (001)-oriented Au substrate comprised of two rigid atomic layers. Figure S14 shows the as-built $MoS_2/MoSe_2/Au$ heterostructure of size $l_{\mathrm{top}}/l_{\mathrm{bot}}/l_{\mathrm{bot}}$ and with a twist angle of θ. In Table S1, we provide the details of all the (36) $MoS_2/MoSe_2/Au$ MD systems analyzed for this investigation.

The TMDC layers are built perfectly flat, where atom positions are located at their equilibrium distance satisfying the lattice parameters predicated by the interatomic potentials (see Ref. [3] for MoS2 and $MoSe_2$), which predict a $MoSe_2/MoS_2$ lattice mismatch, δ, of 0.9639. We set the lattice constant in the Au FCC crystal to 4.08 Å [4]. The initial interlayer separations are chosen in accord with the equilibrium interlayer distance prescribed by the potentials (see Section S10).

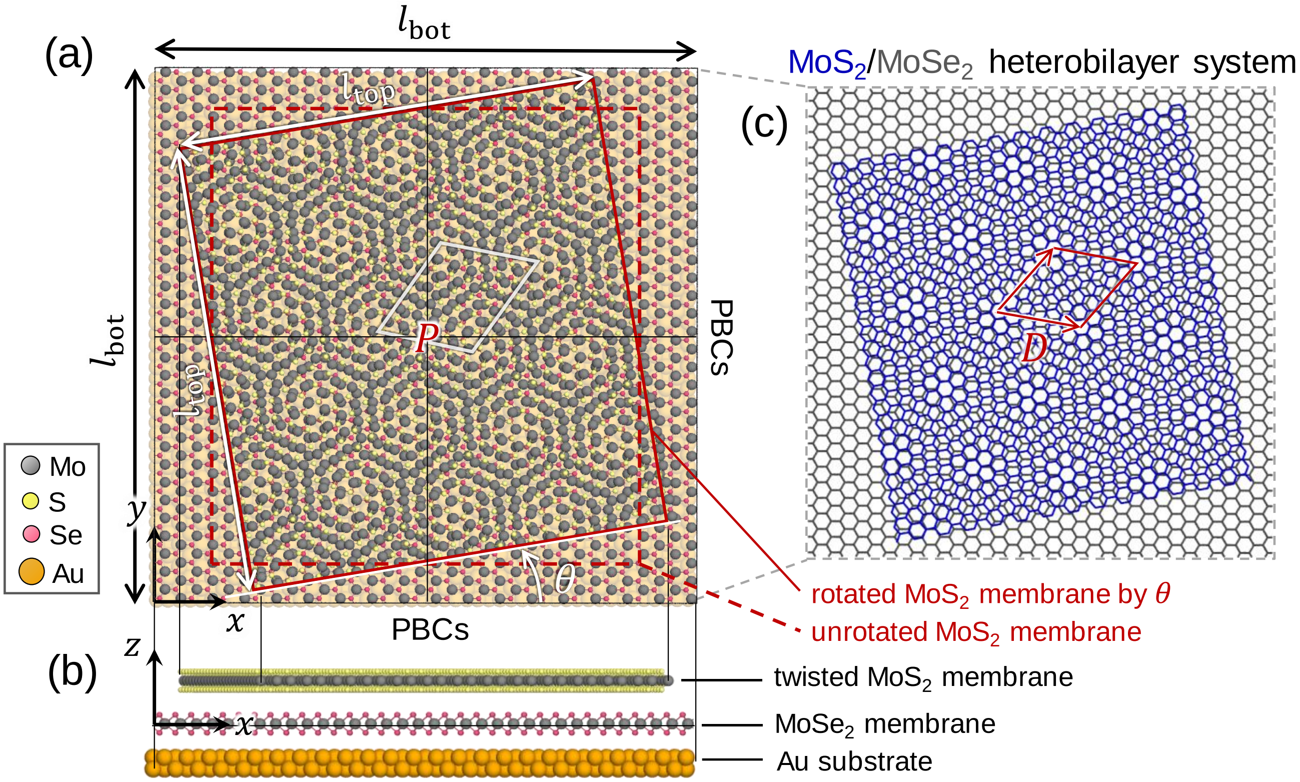

Figure S14. As-built computational domain with periodic boundary conditions (PBCs). (a) Top view and (b) front view of the heterostructure system comprised of a θ-twisted top MoS_2 layer, an intermediate $MoSe_2$ layer, and a bottom Au substrate. (c) Representation of intralayer bonds, which evidences the as-built moiré patterns in the bilayer system. The moiré unit cell is marked in white in (a), and in red in (c). Note that high-θ bilayer configurations lead to small-size moiré periodicity, D.

Table S1. Details of the 36 $MoS_2/MoSe_2/Au$ heterosystems simulated for this investigation.

$MoS_2/MoSe_2/Au$ herosystem size (in* nm*)	***Number of atoms***	***Twist angle, θ***	***Prestrain in the MoS_2***
**80/100/100**	216K/315K/242K	0°	0%, 0.5%, 1%, 1.5%, 2%, 3%, 5%
		0.5°	0%
		1°	0.5%,1%,1.5%,2%
		2°	0%, 2%
		3°	0%, 2%
		5°	0%, 2%
		6°	0%
		7°	0%, 2%
		8°	0%, 2%
		9°	0%
		10.5°	0%
		12°	0%, 2%
		13.5°	0%
**80/120/120**	216K/454K/348K	15°	0%
		17.5°	0%
		20°	0%
		22.5°	0%
		25°	0%
		27.5°	0%
		30°	0%
**240/260/260**	1.94m/2.12m/1.63m	0°	0%
		1°	0%

*Prestraining is only induced in the top MoS_2 membrane along the *armchair* orientation.

S6. FFT analyses of MD MoS_2 topographies as a function of θ

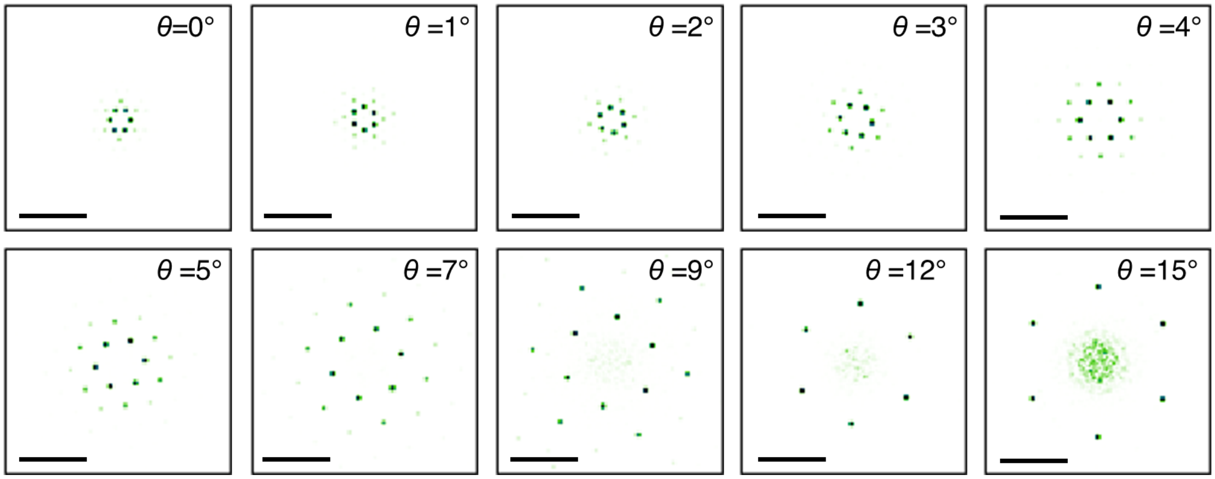

Figure S15. FFT analyses of MD MoS_2 topographies with distinct twist angles, θ. Scale bar is 5 nm^{-1}.

S7. Computation of atomic-level strains

Atomic-level strain tensors in the top MoS_2 layers are calculated using the "atomic strain" modifier featured in the OVITO visualization software [5]. This algorithm allows us to compute the resulting deformation at the atomic sites in the MoS_2 membrane upon relaxation. The modifier cutoff value is set to 10 Å. For the strain calculations, we assume plain strain in the MoS_2 membrane, where $\varepsilon_{xy} = \varepsilon_{yx}$ and $\varepsilon_{zz} = \varepsilon_{xz} = \varepsilon_{yz} = 0$. Then, the atomic Green-Lagrangian strain tensor is fully defined through

$$\mathbf{E} = \begin{pmatrix} \varepsilon_x & \varepsilon_{xy} \\ \varepsilon_{xy} & \varepsilon_y \end{pmatrix} . \tag{S1}$$

We calculate the local *intralayer* strains, $\varepsilon_{\text{local}}$, via the squared sum of the eigenvalues of the strain tensor, **E**, [6]

$$\varepsilon_{\text{local}} = \sqrt{\lambda_1^2 + \lambda_2^2} \ , \tag{S2a}$$

where

$$\lambda_{1,2} = \text{Eig}(\mathbf{E}) = \frac{\varepsilon_x + \varepsilon_y}{2} \pm \left(\left(\frac{\varepsilon_x + \varepsilon_y}{2} \right)^2 - \varepsilon_x \varepsilon_y + \varepsilon_{xy}^2 \right)^{0.5} . \tag{S2b}$$

Alternatively, we also assess in the MoS_2 membranes the value of atomic-level maximum strains, ε_{max}, whose analytical relationship with the components of tensor **E** from Eq. (S1)–assuming plain strain conditions–is defined by

$$\varepsilon_{\text{max}} = \frac{\varepsilon_x + \varepsilon_y}{2} + \sqrt{\left(\frac{\varepsilon_x + \varepsilon_y}{2} \right)^2 + \left(\frac{\varepsilon_{xy}}{2} \right)^2} \ . \tag{S3}$$

Figure S16 shows the topography (left-hand side pictures) and the corresponding $\varepsilon_{\text{local}}$ and ε_{max} maps of unstrained (Fig. S16(a)) vs. pre-strained (Fig. S16(b)) MoS_2 membranes with $\theta = 1^{\text{o}}$. To obtain an optimal definition in the strain maps of the MoS_2 membranes, the atomic-level deformations are computed taking the initial and final atom coordinates from as-built and the relaxed configurations of the heterosystem, respectively. It is important to mention that we capture

the relaxed state of the MoS_2 membranes using atom coordinates averaged over 5 ps. This leads to a smooth deformation field computed by means of the OVITO's modifier.

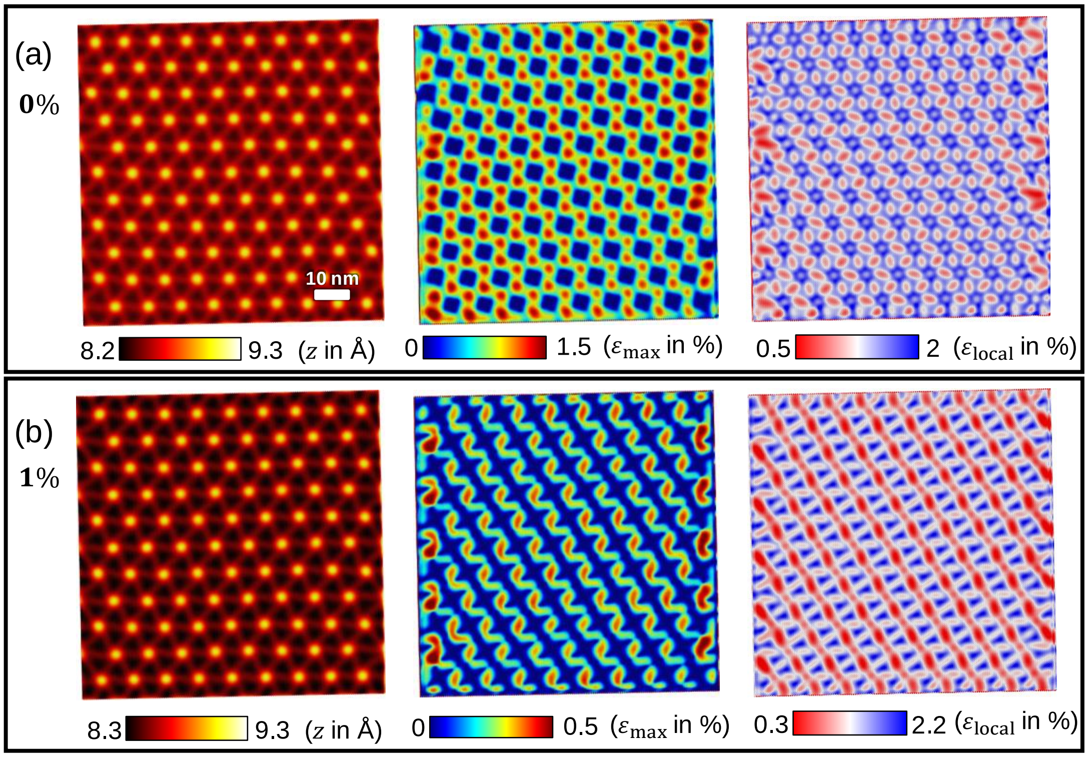

Figure S16. Atomic-level topography and strain maps of MoS_2 after relaxation. The $MoS_2/MoSe_2/Au$ heterosystem is of size 80/100/100 nm with a twist angle of 1°. (a) Unstrained MoS_2 and (b) 1% pre-strained MoS_2.

In addition, Fig. S17 displays the ε_{local} maps in both MoS_2 (top) and $MoSe_2$ (bottom) with the absence (Fig. S17(a)) and presence (Fig. S17(b)) of prestraining in MoS_2. These results are indicative of the influence of prestraining on the strain levels and patterns. With increasing prestrain levels, a local raise in the ε_{local} levels is found in both membranes predominantly across non-AA stacking zones. This also leads to a change in the ε_{local} patterns as compared with those from non-prestrained $MoS_2/MoSe_2$.

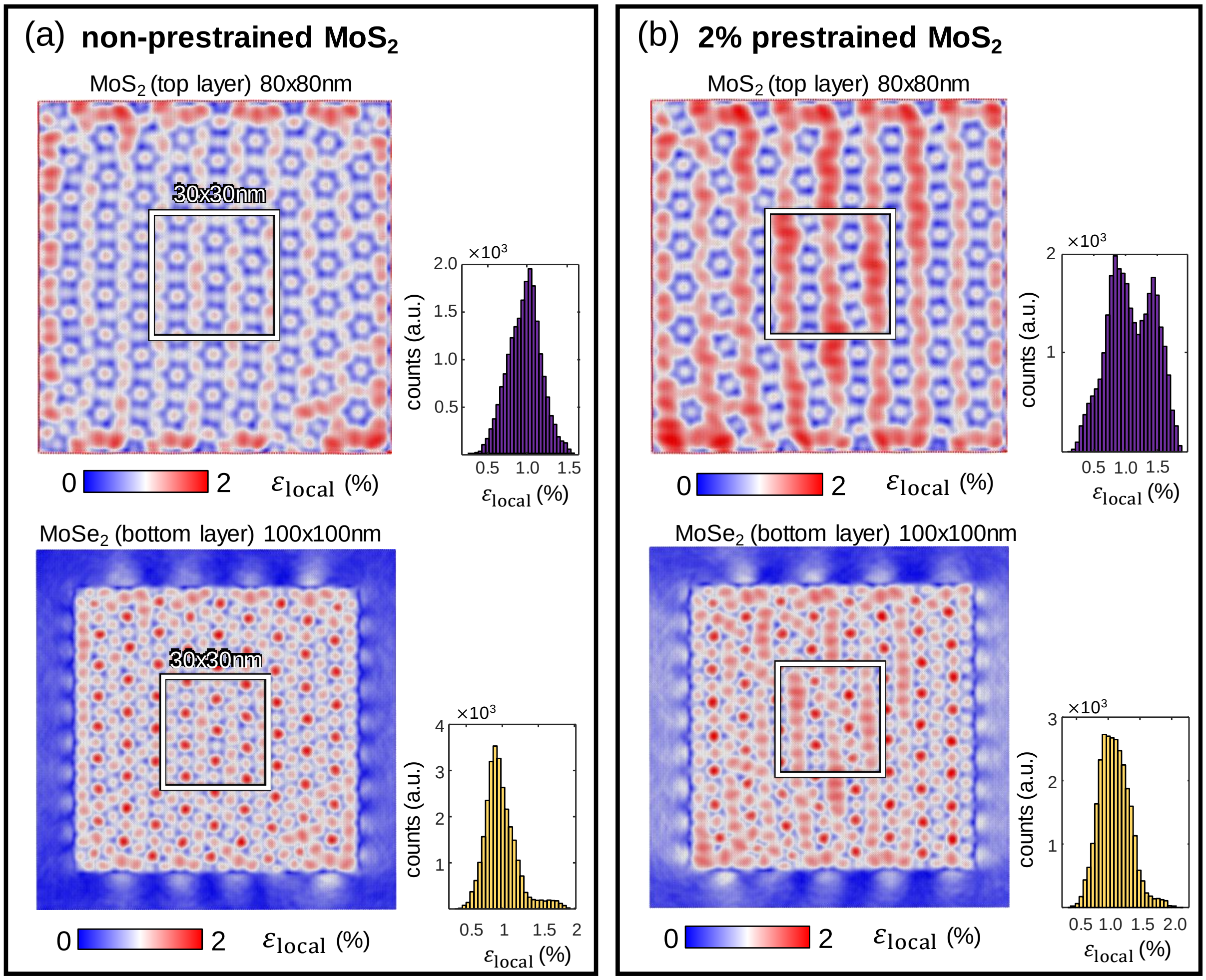

Figure S17. Atomic-level strain maps of MoS_2 and $MoSe_2$ after relaxation. The $MoS_2/MoSe_2/Au$ heterosystem is of size 80/100/100 nm with a twist angle of 0º. (a) Non-prestrained and (b) 2% prestrained MoS_2. The ε_{local} distributions are extracted from the 30x30-nm^2 areas marked with a square.

S8. Atomic displacements in reconstructed MoS_2

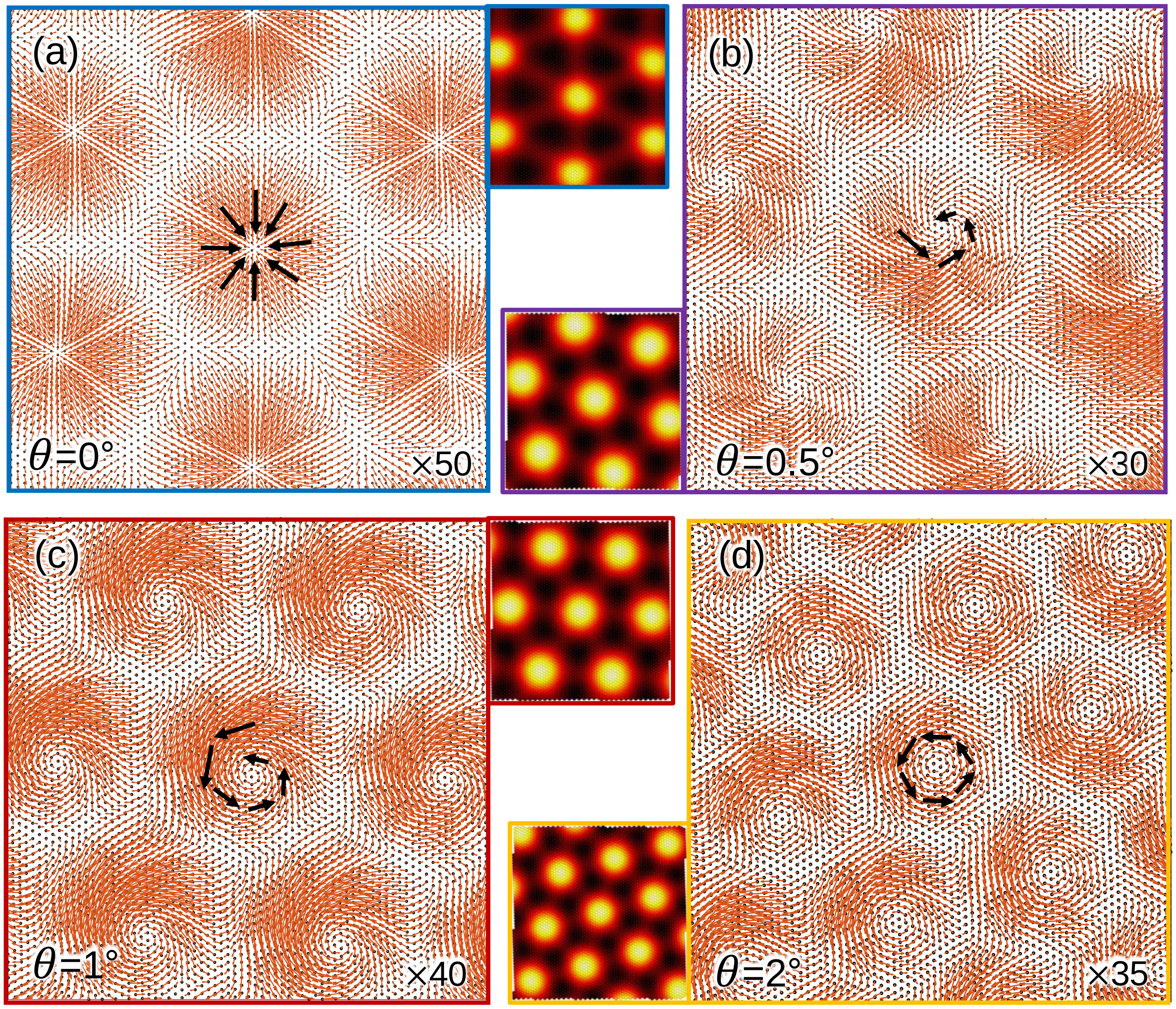

Figure S18. Vector fields of Mo displacements (in MoS_2) extracted from twisted MoS_2/$MoSe_2$/Au heterosystems under small twist angles, θ. The points represent the positions of single Mo atoms in the relaxed MoS_2. Individual displacement vectors are depicted in red and scaled as marked on the bottom-right side of the figures. The Mo displacement vectors are calculated using the atomic positions from the as-built (initial) and the relaxed (final) MD membranes using averaged coordinates over 5 ps. The analyzed MoS_2 areas have a size of 20×20 nm^2. The insets provide the corresponding MoS_2 topographies. For guidance, the black arrows mark the direction of the displacements around AA sites.

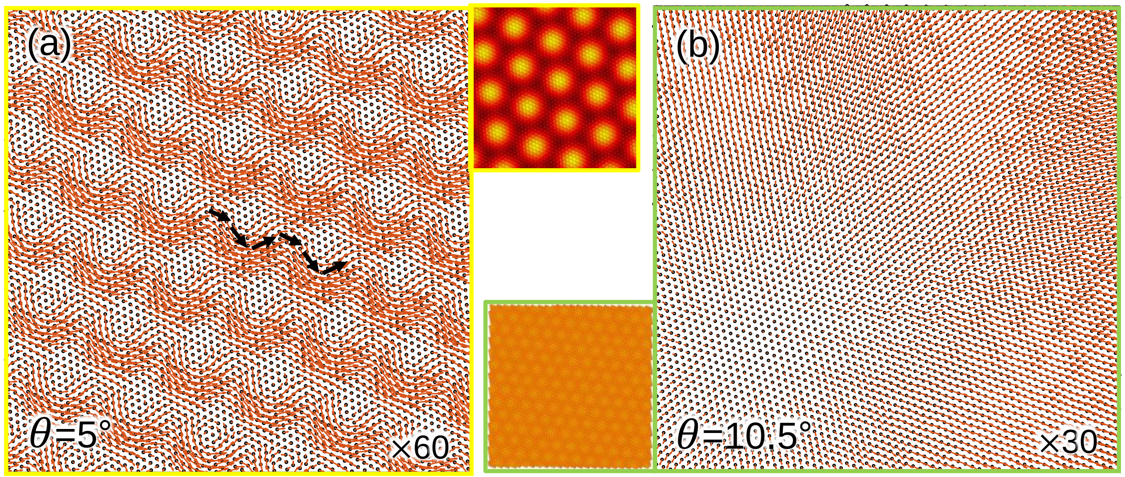

Figure S19. Vector fields of Mo displacements (in MoS_2) extracted from twisted $MoS_2/MoSe_2/Au$ heterosystems under (a) a low twist angle and (b) a high twist angle. The points represent the positions of single Mo atoms in the relaxed MoS_2. Individual displacement vectors are depicted in red and scaled as marked on the bottom-right side of the figures. The Mo displacements are calculated using the atomic positions from the as-built (initial) and the relaxed (final) MD membranes using averaged coordinates over 5 ps. The analyzed MoS_2 areas have a size of 20×20 nm^2. The insets provide the corresponding MoS_2 topographies. For guidance, the black arrows in (a) mark the direction of the Mo displacements.

S9. The effect of the Grüneisen parameter on the simulated Raman spectra

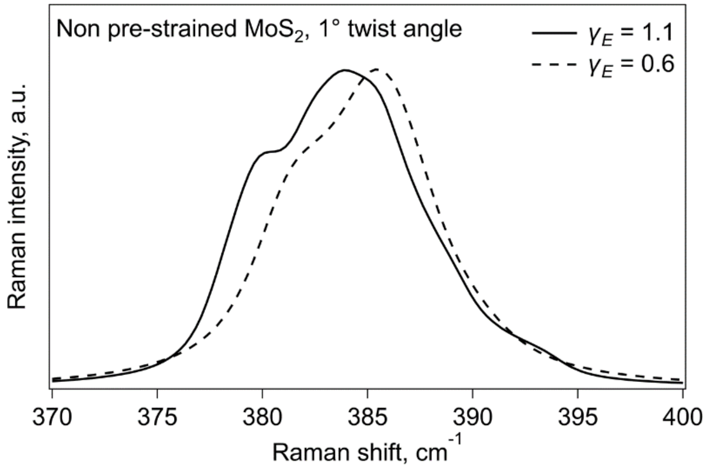

Figure S20. Simulated Raman spectra as a function of the Grüneisen parameter, γ_{E}, (cf. Eq. (2) in the main text) as calculated from MD results of a non-prestrained 30×30-nm MoS_2 membrane with a twist angle of $\theta = 1°$.

S10. Empirical potentials for interlayer interactions

Van der Waals (vdW) forces fundamentally govern the relatively weak interlayer bonding between multilayer systems. In particular, attaching TMDC layers gives rise to steric effects in the resulting interlayer interactions. This leads to a strong energy dependence in terms of the stacking sequence of adjacent layers. A clear understanding of the atomic-scale displacements involved in the development of stacking domains becomes pivotal for the study of the governing processes associated to the formation of moiré superlattices in bilayer systems [7]. Large-scale atomistic simulations of such nanomechanical systems are well beyond the capabilities of first-principles methods. Thus, reliable empirical potentials must be applied in the aim of replacing computationally expensive DFT calculations with classical force-field (molecular dynamics) simulations.

The archetypal Lennard-Jones (LJ) model estimates the vdW coupling by considering the pair-wise interaction between atoms with an $1/r^6$ attracting term, where the potential energy between two atoms separated by a pair-wise distance r_{ij} is defined through

$$V(r_{ij}) = 4\epsilon\left(\frac{\sigma^{12}}{{r_{ij}}^{12}} - \frac{\sigma^{6}}{{r_{ij}}^{6}}\right). \qquad \text{(S4)}$$

Here, ϵ and σ are fitting parameters. Equation (S4) predicts the absolute energy minimum of the potential well –satisfying $\mathrm{d}V(r_{ij})/\mathrm{d}r_{ij} = 0$– at a distance $r_{\mathrm{eq}} = 2^{1/6}\sigma$. With $r_{ij} > r_{\mathrm{eq}}$ the potential energy smoothly vanishes towards 0; where $V(r_{ij}) = 0$ is strictly set after a cutoff radius [8]. Table S2 provides the LJ coefficients [9] used to compute the interlayer vdW-like forces in our $MoS_2/MoSe_2/Au$ heterosystems.

We invariably adopt the LJ model to prescribe the vdW coupling between the $MoSe_2$ layer and the Au substrate (Mo-Au and Se-Au interaction pairs, which prescribes an interlayer spacing of 3.403 Å). By recourse to the continuum model described by Zhu et al. [10], we estimate a cohesive energy between the $MoSe_2$ and Au substrate of ≈-190 $\mathrm{mJ/m^2}$, which accounts for a layer-substrate interaction with similar adhesion levels to those measured in experiments of TMDC monolayers deposited on highly adhesive substrates; see, e.g., Ref. [11].

Table S2. LJ parameters used in present MD simulations.

	Mo-Au	*Se-Au*	*Mo-Mo*	*Mo-Se*	*Mo-S*	*Se-S*
σ (Å)	3.567	3.032	2.719	3.192	3.126	3.670
ϵ (meV)	4.78038	23.2495	2.432	5.543	5.379	12.262

Although the LJ potential has been largely employed to prescribe the adhesion between layers using MD simulations [10, 12, 13], this isotropic model happens to be too simplistic to describe variations in the relative alignment of adjacent layers, where possible increases in the vdW contribution are due to corrugation-vs-sliding processes that occur as a function of the local stacking sequence [14]. Along these lines, the formation of moiré patterns in bilayer components –which has a strong dependence on the interlayer stacking– can possibly be hindered using the LJ model.

To account for this stacking dependence, Kolgomorov and Crespi (KC) intentionally developed an empirical potential to prescribe realistic interlayer interactions in graphite (composed of graphene layers) [15]. The potential contains an $1/r^6$ vdW attraction term (as in the LJ model) and an exponentially decaying repulsion term that accounts for interlayer overlaps, where the interlayer cohesion is essentially prescribed by a combination of long-range vdW forces and short-range orbital overlap contributions. To reflect the overlap directionality, KC introduced a function, f, which rapidly decays with the transverse distance ρ. The form of the potential is given by

$$V(r_{ij}) = \mathrm{e}^{-\lambda(r_{ij}-z_0)}\, V_{\mathrm{p}} - A\left(\frac{r_{ij}}{z_0}\right)^{-6}, \tag{S5}$$

where $V_{\mathrm{p}} = [C + f(\rho_{ij}) + f(\rho_{ji})]$; $\rho_{ij}^2 = r_{ij}^2 - (\mathbf{r}_{ij}\cdot\mathbf{n}_i)^2$, $\rho_{ji}^2 = r_{ji}^2 - (\mathbf{r}_{ji}\cdot\mathbf{n}_j)^2$, and $f(\rho) = \mathrm{e}^{-(\rho/\delta)^2}\sum_{n=0}^{2} C_{2n}(\rho/\delta)^{2n}$.

Note in Eq. (S5) that the potential consists of eight fitting parameters for each type of pair interaction; namely $C, C_0, C_2, C_4, \delta, \lambda, A$, and the scaling factor z_0. The KC potential includes the stacking dependence in the V_{p} term which critically determines the energy barrier to shear one layer with respect to the other in a bilayer system. Notice that, in similar terms to the LJ model, the KC potential is also set to 0 after a cutoff radius. Energies decay sufficiently fast, thus enabling a continuous $V(r_{ij})$ shift to 0 at the cutoff. It is important to impose a sufficiently large cutoff to

ensure smooth decaying forces and to include all the pairs to build the neighbor lists for the calculation of the normals ($\mathbf{n}_i$ and $\mathbf{n}_j$); cf. Fig. 5 in Ref. [16].

To model the interactions between the MoS_2 and the $MoSe_2$ layers, we employ in our MD simulations the KC potential from Ref. [16], which includes the parametrization of the KC function for TMDC interlayer interactions. To estimate the equilibrium distance between the MoS_2 and the $MoSe_2$ layers that predicts the parametrized KC potential for MoS_2 and $MoSe_2$ interlayer interactions, we run a complementary molecular statics simulation containing a 40×40-nm MoS_2/$MoSe_2$ bilayer system (with perfectly flat membranes) that gradually changes the interlayer separation (from 10 Å to 5 Å). The results from this simulation indicate that a minimum energy state is attained with an interlayer separation of ≈3.2 Å; see Fig. S21.

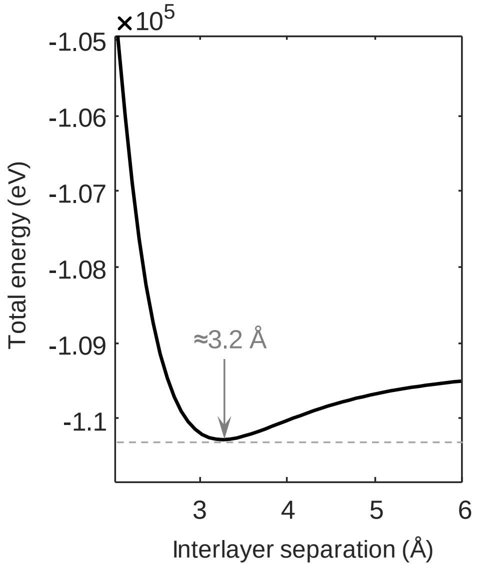

Figure S21. Total energy as a function of the MoS_2–$MoSe_2$ interlayer separation as predicted by the KC potential parametrized by Naik et al. [16].

S11. The influence of the interlayer empirical potential on lattice reconstruction

To test the ability of the above empirical potentials of predicting the interlayer stacking associated to lattice reconstruction, we conduct a systematic analysis of MD simulations with MoS_2/$MoSe_2$/Au heterosystems of size 80/100/100 nm, where the MoS_2-$MoSe_2$ interlayers are separately described by LJ and KC potentials. To this end, we assume the LJ potential under the (Mo-S, Mo-Mo, Mo-Se, S-Se) pair parameters given in Table S2 (cf. the "LJ" simulation results

given in Figs. S22(b) and S23(c) with $\theta = 0.5°$ and $\theta = 0°$, respectively) as well as the KC potential parametrized by Naik et al. [16] (cf. the "KC" simulation results given in Figs. S22(a) with $\theta = 0.5°$ and S23(a) with $\theta = 0°$). Special attention is given to the resulting relaxed states of the top MoS_2 membranes in relation to the employed MoS_2-$MoSe_2$ interlayer model.

The atomic-resolution MoS_2 topographies in Fig. S17 reveal that both empirical potentials can predict the patterning of out-of-plane displacements throughout the top layers. These patterns systematically correspond with specific stacking domain sites, as shown in Fig. S17(b). The "KC" results from Fig. S23(a) show a good correlation with the STEM image of twisted bilayer graphene from Fig. 1(g) in Ref. [17]. In our simulations, the maximum height points in the topographies mark indistinguishably the location of the AA main sites in our relaxed MoS_2 membranes. These domains generate the most marked out-of-plane deformations. In contrast, the AB and BA sites are located at the minimum heights in the topography maps. For reference of the MoS_2-$MoSe_2$ stacking terminology, see the inset to Fig. S23(b). While the above holds for both empirical interlayer models, the MoS_2 topographies from the "KC" simulations exhibit more pronounced out-of-plane corrugations around the AA sites than in those from the "LJ" simulations. With greater levels of adhesion (this is easily achieved by increasing LJ parameter ϵ [18]), we observe in our "LJ" simulations that the amplitudes of the AA-related corrugations remain roughly unaffected.

Although the stacking domains found in both "KC" and "LJ" simulations exhibit similar patterning in terms of the relative areas of the domains, the former simulations predict slightly narrower AA stacking sites and larger AB and BA domains than those from the latter. In the "KC" simulations, stacking domain formation is then favored by the enhanced interlayer sliding as a result of the V_{p} term of the KC potential; see Eq. (S5).

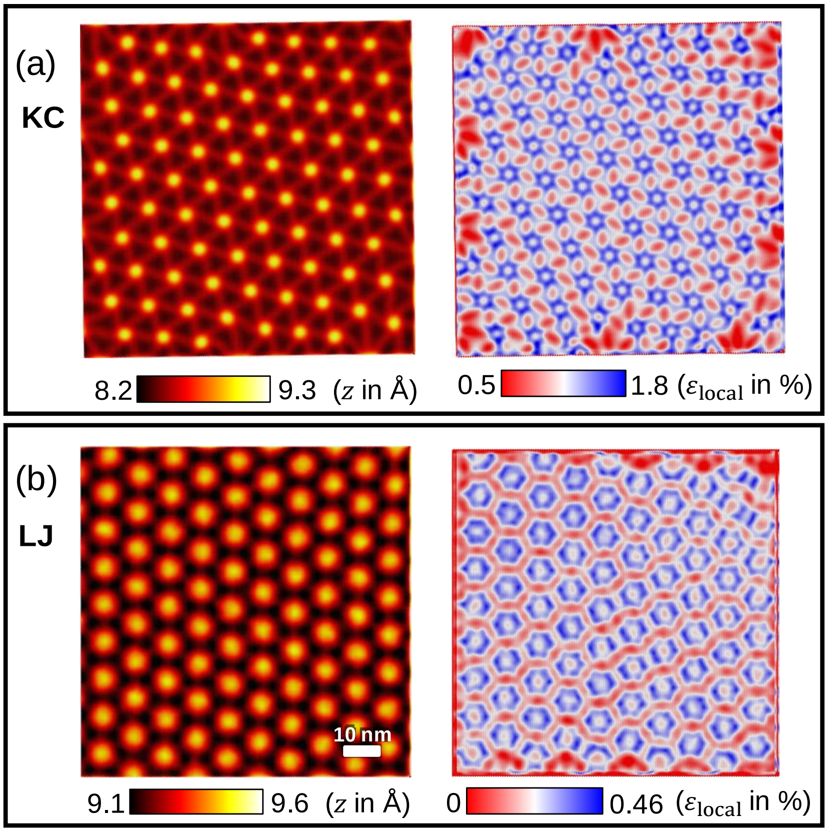

Figure S22. Influence of the MoS_2–$MoSe_2$ interlayer potential on the resulting MoS_2 topography and $\varepsilon_{\text{local}}$ maps after relaxation. The MoS_2/$MoSe_2$/Au heterosystem of size 80/100/100 nm with a twist angle of 0.5º. MoS_2–$MoSe_2$ interlayer interactions defined by (a) the LJ potential (Eq. (S5)) with the parameters from Table S2 and by (b) the parametrized KC potential from Ref. [16].

The outcomes from our simulations suggest that the "KC" simulations generate, in particular, a more complex $\varepsilon_{\text{local}}$ strainscape than the "LJ" counterparts. Also, the atom-level strain analyses from the former exhibit the much greater $\varepsilon_{\text{local}}$ levels throughout the relaxed MoS_2 membranes than those obtained in the "LJ" simulations; compare the strain maps in Fig. S22(a, b). These results suggest that the KC potential prescribes bilayer relaxations that involve a major interlayer sliding, which allows for the energetically favored accommodation of the moiré lattices (see Section S10). Along these lines, the $\varepsilon_{\text{local}}$ map given in Fig. S22(a) shows a good agreement with the FEM-DFT results reported in Fig. 6(d) from Ref. [19], which supports the above argument.

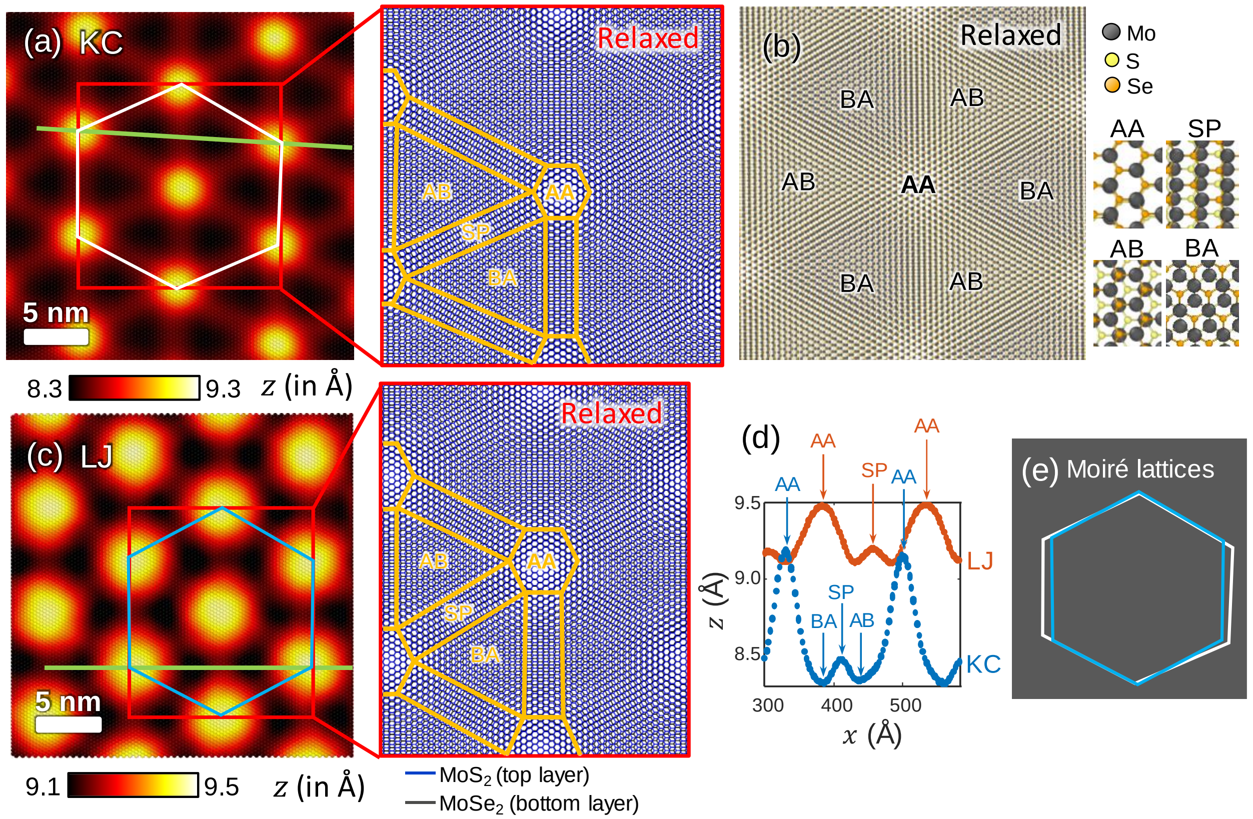

Figure S23. Lattice reconstruction with the KC and LJ potentials describing the interlayer interaction between MoS_2 and $MoSe_2$. The $MoS_2/MoSe_2/Au$ heterosystem is of 80/100/100 nm in size, with a twist angle of 0°. Topography maps of a relaxed 30×30-nm^2 MoS_2 membrane using (a) the KC potential and (c) the LJ potential for the MoS_2–$MoSe_2$ interactions. The insets to these figures mark the specific stacking domains formed during relaxation. (b) Stacking domains in the relaxed MoS_2–$MoSe_2$ system from the "KC" simulation. (d) Out-of-plane displacements from the cuts marked in green in (a) and (c). For comparison. the relaxed moiré hexagonal cells highlighted in (a) and (c) for KC and LJ interlayer potentials, respectively, are brought together in (e).

Considering these results, it is clear that the parametrized KC potential from Ref. [16] overperforms the LJ potential when it comes to predicting the deformation states of twisted bilayer TMDC systems. Moreover, in view of the DFT results reported in Ref. [20] which emphasize that the deformations of the top layer in twisted bilayer systems fundamentally governs the properties of moiré superlattices as a result of stacking-dependent interlayer interactions, the prediction of realistic local stacking and out-of-plane deformations in our simulations using the KC potential becomes pivotal for the comparison of the MD moiré patterns with those observed in our $MoS_2/MoSe_2$ experiments.

S12. Interlayer coupling in MoS_2/$MoSe_2$ heterostructures

We evaluate the total energy of the system of atoms that comprise relaxed, twisted MoS_2 membranes in terms of the twist angle θ. The plot in Fig. S18(b) shows the evolution of the absolute value of the MoS_2 total energy with θ, where a minimum energy state is reached under low-angle configurations (from $\theta = 0°$ to $\theta = 1°$). Increases in the twist angle lead to a gradual buildup in energy levels (from $\theta = 2°$ to $\theta = 12°$), while energy values stabilize for angles from $\theta = 12°$ to $\theta = 30°$. These maximum-energy states of MoS_2 are related to twisted bilayer configurations that prevent the development of moiré superlattices (see Fig. 4 in the main text).

These results agree with the view that interlayer coupling strength decreases with θ [20]. In addition to the discussion given in Sections S10 and S11, the plots from Fig. S24 indicate that the "KC" simulations can predict the influence of θ on the resulting MoS_2-$MoSe_2$ interlayer interactions more accurately than the "LJ" simulations (Fig. S24(a)), where the latter simulations are more tentative when it comes to reproducing a smooth low-angle-to-high-angle transition in terms of the interlayer coupling.

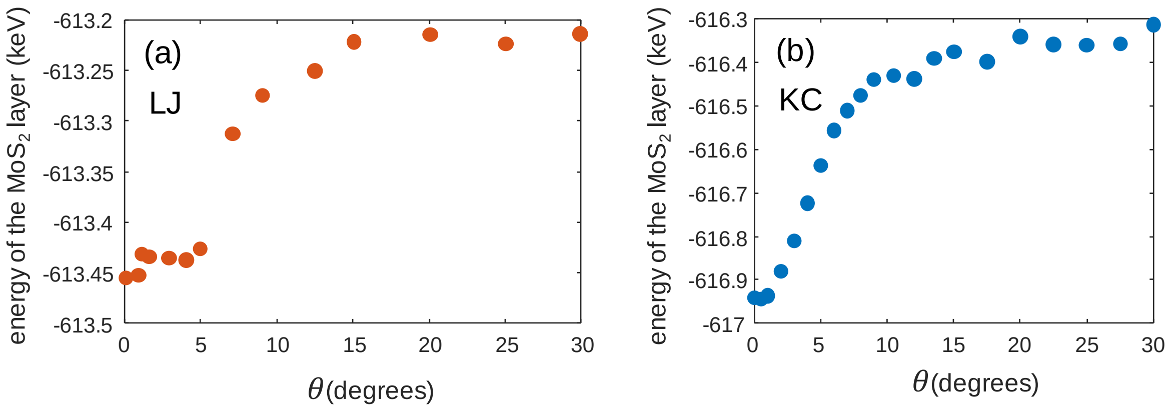

Figure S24. Total energy of MoS_2 as a function of the twist angle θ in MoS_2/$MoSe_2$/Au heterosystems with 80×80-nm twisted MoS_2 membranes. We compare the results from the MD simulations that employ the LJ (a) vs. KC (b) potential models to describe the MoS_2–$MoSe_2$ interlayer interactions.

References

[1] K. Xu, W. Sun, Y. Shao, F. Wei, X. Zhang, W. Wang and P. Li, "Recent development of PeakForce Tapping mode atomic force microscopy and its applications on nanoscience," *Nanotechnology Reviews*, vol. 7, pp. 605-621, 2018.

[2] A. Rodriguez, M. Kalbáč and O. Frank, "Strong localization effects in the photoluminescence of transition metal dichalcogenide heterobilayers," *2D Materials*, vol. 8, p. 025028, 2 2021.

[3] A. Kandemir, H. Yapicioglu, A. Kinaci, T. Çağın and C. Sevik, "Thermal transport properties of MoSsub2/suband MoSesub2/submonolayers," *Nanotechnology*, vol. 27, p. 055703, 1 2016.

[4] X. W. Zhou, R. A. Johnson and H. N. G. Wadley, "Misfit-energy-increasing dislocations in vapor-deposited CoFe/NiFe multilayers," *Phys. Rev. B*, vol. 69, no. 14, p. 144113, 4 2004.

[5] A. Stukowski, "Visualization and analysis of atomistic simulation data with OVITO–the Open Visualization Tool," *Model. Simul. Mat. Sci. Eng.*, vol. 18, p. 015012, 12 2009.

[6] J. Quan, L. Linhart, M.-L. Lin, D. Lee, J. Zhu, C.-Y. Wang, W.-T. Hsu, J. Choi, J. Embley, C. Young, T. Taniguchi, K. Watanabe, C.-K. Shih, K. Lai, A. H. MacDonald, P.-H. Tan, F. Libisch and X. Li, "Phonon renormalization in reconstructed MoS2 moiré superlattices," *Nat. Mater.*, vol. 20, pp. 1100-1105, 8 2021.

[7] C. N. Lau, M. W. Bockrath, K. F. Mak and F. Zhang, "Reproducibility in the fabrication and physics of moiré materials," *Nature*, vol. 602, pp. 41-50, 2 2022.

[8] M. P. Allen and D. J. Tildesley, Computer Simulation of Liquids, 2nd ed., O. U. Press, Ed., 2017.

[9] A. K. Rappe, C. J. Casewit, K. S. Colwell, W. A. Goddard and W. M. Skiff, "UFF, a full periodic table force field for molecular mechanics and molecular dynamics simulations," *J. Am. Chem. Soc.*, vol. 114, pp. 10024-10035, 1992.

[10] S. Zhu and T. Li, "Wrinkling instability of graphene on substrate-supported nanoparticles," *J. Appl. Mech,* vol. 81, 2 2014.

[11] D. Lloyd, X. Liu, N. Boddeti, L. Cantley, R. Long, M. L. Dunn and J. S. Bunch, "Adhesion, Stiffness, and Instability in Atomically Thin MoS2 Bubbles," *Nano Lett.,* vol. 17, pp. 5329-5334, 9 2017.

[12] Y. Guo and W. Guo, "Soliton-like thermophoresis of graphene wrinkles," *Nanoscale,* vol. 5, no. 1, pp. 318-323, 2013.

[13] L. Peng, H. Chan, P. Choo, T. W. Odom, S. K. R. S. Sankaranarayanan and X. Ma, "Creation of Single-Photon Emitters in WSe(2) Monolayers Using Nanometer-Sized Gold Tips.," *Nano Ltt.,* vol. 20, no. 8, pp. 5866-5872, 8 2020.

[14] Z. Kozioł, G. Gawlik and J. Jagielski, "Van der Waals interlayer potential of graphitic structures: From Lennard–Jones to Kolmogorov–Crespy and Lebedeva models," *Chin. Phys. B,* vol. 28, p. 096101, 9 2019.

[15] A. N. Kolmogorov and V. H. Crespi, "Registry-dependent interlayer potential for graphitic systems," *Phys. Rev. B,* vol. 71, no. 23, p. 235415, 6 2005.

[16] M. H. Naik, I. Maity, P. K. Maiti and M. Jain, "Kolmogorov–Crespi Potential For Multilayer Transition-Metal Dichalcogenides: Capturing Structural Transformations in Moiré Superlattices," *J. Phys. Chem. C,* vol. 123, pp. 9770-9778, 2019.

[17] A. Kerelsky, L. J. McGilly, D. M. Kennes, L. Xian, M. Yankowitz, S. Chen, K. Watanabe, T. Taniguchi, J. Hone, C. Dean, A. Rubio and A. N. Pasupathy, "Maximized electron interactions at the magic angle in twisted bilayer graphene," *Nature,* vol. 572, pp. 95-100, 8 2019.

[18] J. Varillas and O. Frank, "Wrinkle development in graphene sheets with patterned nano-protrusions: A molecular dynamics study," *Carbon,* vol. 173, pp. 301-310, 2021.

[19] N. P. Kazmierczak, M. Van Winkle, C. Ophus, K. C. Bustillo, S. Carr, H. G. Brown, J. Ciston, T. Taniguchi, K. Watanabe and D. K. Bediako, "Strain fields in twisted bilayer graphene," *Nature Materials,* vol. 20, pp. 956-963, 7 2021.

[20] W. T. Geng, V. Wang, J. B. Lin, T. Ohno and J. Nara, "Angle Dependence of Interlayer Coupling in Twisted Transition Metal Dichalcogenide Heterobilayers," *J. Phys. Chem. C,* vol. 125, pp. 1048-1053, 1 2021.

[21] M. Zhang, G. H. Tang, Y. F. Li, B. Fu and X. Y. Wang, "Phonon Thermal Properties of Heterobilayers with a Molecular Dynamics Study," *Int. J. Thermophys.,* vol. 41, 2020.

[22] C. Desgranges and J. Delhommelle, "Evaluation of the grand-canonical partition function using expanded Wang-Landau simulations. III. Impact of combining rules on mixtures properties," *The Journal of Chemical Physics,* vol. 140, p. 104109, 2014.
